# Secretion of functional interferon by the type 3 secretion system of enteropathogenic *Escherichia coli*

**DOI:** 10.1186/s12934-024-02397-y

**Published:** 2024-06-01

**Authors:** Irina Rostovsky, Uri Wieler, Alona Kuzmina, Ran Taube, Neta Sal-Man

**Affiliations:** The Shraga Segal Department of Microbiology, Immunology, and Genetics, Faculty of Health Sciences, Ben-Gurion University of the Negev, P.O. Box 653, 84105 Beer-Sheva, Israel

**Keywords:** Antiviral, Antiproliferation, Interferon, Oral drug delivery, Protein secretion, Type III secretion system

## Abstract

**Background:**

Type I interferons (IFN-I)—a group of cytokines with immunomodulatory, antiproliferative, and antiviral properties—are widely used as therapeutics for various cancers and viral diseases. Since IFNs are proteins, they are highly susceptible to degradation by proteases and by hydrolysis in the strong acid environment of the stomach, and they are therefore administered parenterally. In this study, we examined whether the intestinal bacterium, enteropathogenic *Escherichia coli* (EPEC), can be exploited for oral delivery of IFN-Is. EPEC survives the harsh conditions of the stomach and, upon reaching the small intestine, expresses a type III secretion system (T3SS) that is used to translocate effector proteins across the bacterial envelope into the eukaryotic host cells.

**Results:**

In this study, we developed an attenuated EPEC strain that cannot colonize the host but can secrete functional human IFNα2 variant through the T3SS. We found that this bacteria-secreted IFN exhibited antiproliferative and antiviral activities similar to commercially available IFN.

**Conclusion:**

These findings present a potential novel approach for the oral delivery of IFN via secreting bacteria.

**Supplementary Information:**

The online version contains supplementary material available at 10.1186/s12934-024-02397-y.

## Background

Type I interferons (IFNs) are cytokines with immunoregulatory roles associated mainly with antiviral responses, adaptive immunity, and antiproliferative effects on immune and non-immune cells [1]. In humans, the various type I IFNs induce their biological effects through a common receptor known as the type I IFN receptor (IFNAR), a heterodimer composed of two membrane proteins, designated IFNAR1 and IFNAR2. Upon binding of the type I IFN to the IFNAR heterodimer, the associated tyrosine kinases, Janus-activated kinase 1 (Jak1) and tyrosine kinase 2 (Tyk2), are activated, and they, in turn, phosphorylate the signal transducer and activator of transcription 1 (STAT1) and 2 (STAT2) proteins. The phosphorylated STAT1-STAT2 heterodimer interacts with IFN-regulatory factor 9 (IRF9), which is then translocated into the nucleus to initiate the transcription of IFN-stimulated genes (ISGs). Expression of ISGs primes both immune and non-immune cells toward the successful resolution of infection or stress. Some ISGs are considered robust genes that can be induced by small amounts of low-binding IFNs, while others require high concentrations of high-affinity IFNs and high concentrations of cell surface receptors [2–4]. The former group includes many antiviral response genes, while the latter comprises immunomodulatory and antiproliferation response genes.

Although type I IFNs share high sequence homology and have a common receptor, they induce different cellular activities at various intensities, affecting diverse cell types. For example, IFNβ elicits a significantly higher antiproliferative response than IFNα in cancerous cells, and it is also the only type I IFN that has been used successfully for treating multiple sclerosis [5–8]. Similarly to IFNβ, other IFNs have become attractive targets for drug development due to their antiviral and immune-modulation activities. Their clinical effectiveness has been successfully established for various human diseases, including hepatitis C, relapsing forms of multiple sclerosis, and certain types of cancer [9–12]. In addition, IFN therapy has recently entered clinical trials for COVID-19 [13, 14]. However, as type I IFNs are proteins, their oral administration is challenging due to their susceptibility to degradation by the strong acid environment and the proteolytic enzymes in the stomach. Therefore, IFN-based drugs are currently administrated parenterally, mostly subcutaneously, but also intravenously. Since IFN administered by these routes has a short circulation half-life, multiple injections per week for prolonged periods may be required. Patient compliance with this regimen may be poor due to side effects reported for this treatment, such as erythema and induration at the injection site, as well as rash, pruritus, alopecia, and lichen planus [15, 16].

In this work, we explored the concept of delivering IFN orally by generating IFN-secreting bacteria. We used a mutant version of IFNα2 termed IFN_YNS_, which contains the mutations H57Y, E58N, and Q61S. This recombinant IFN was shown to have enhanced binding affinity to IFNAR, similar to IFNβ, and hence increased biological potency manifested in enhanced antiproliferative and antiviral activities [2]. To facilitate oral delivery of IFN_YNS_, we exploited the type III secretion system (T3SS) of enteropathogenic *Escherichia coli* (EPEC). We chose this particular bacterial secretion system since EPEC survives the harsh conditions of the stomach and, upon reaching the small intestine, expresses the T3SS, which is then used to translocate effector proteins across the bacterial envelope and into eukaryotic host cells [17]. This translocation is achieved by passing through the T3SS apparatus, a syringe-like structure, anchored within the bacterial membranes, with a long extracellular needle that extends toward the host cell membrane. It was previously demonstrated that EPEC, like other T3SS-containing bacteria, can deliver heterologous proteins into diverse cell types [18–22] or secrete them to the culture medium [23–27]. Most of these proteins were reporter proteins, facilitating the study of bacterial translocation mechanisms.

To exploit the T3SS for delivering a heterologous substrate, such as IFN_YNS_, the target protein should be fused to a T3S-like signal peptide at its N-terminus. Although a conserved T3S signal peptide has not been identified [18, 20, 28], it has been shown that fusing reporter proteins, such as TEM-1 beta-lactamase, to the 50 N-terminal amino acid sequence of EspB, a T3SS translocator of EPEC, enables the translocation of the fused proteins from EPEC into the host cells [28]. Therefore, we used this amino acid sequence to facilitate the delivery of IFN_YNS_ via the EPEC T3SS. To promote the secretion of IFN_YNS_ into the extracellular environment upon arriving in the small intestine and not its injection into host cells, we utilized an EPEC Δ*sepD* mutant strain, which has dysregulated type III secretion [28, 29]. This strain is deficient in translocating T3SS proteins into host cells and exhibits an hypersecretion activity of T3SS cargo to the extracellular medium [29]. This mutant strain was reported to be severely attenuated in its ability to infect HeLa cells [30], and its corresponding mutant of the related murine pathogen, *Citrobacter rodentium*, is avirulent in a mouse model [31]. Thus, it can be used as an attenuated bacterium with functional T3SS that imposes a low risk for host colonization and infection.

Here, we report the successful expression of IFN_YNS_ in EPEC and its secretion into the growth medium of the bacteria grown under T3SS-inducing conditions (simulating the small intestine environment). More importantly, we explored the ability of the EPEC-secreted IFN_YNS_ to function correctly (upregulate the transcription of ISGs and promote antiviral and antiproliferation activities in target cells). Our results suggest that our platform produces functional IFN protein that can be further developed as an oral delivery system of IFN.

## Methods

### Bacterial cultivation

WT EPEC O127:H6 strain E2348/69 [streptomycin-resistant] [32], the EPEC null Δ*escN* mutant, which is T3SS deficient, and the EPEC null Δ*sepD* mutant, which hyper-secretes effectors [29, 33] were used to determine IFN_YNS_ secretion through the T3SS of EPEC (Table 1). *Citrobacter rodentium* DBS100 was used to determine IFN_YNS_ secretion via the T3SS of the murine pathogen (Table 1). The bacteria were grown at 37°C in a Luria–Bertani (LB) broth or Dulbecco's Modified Eagle Medium (DMEM, Gibco) supplemented with carbenicillin (100 μg/mL) and streptomycin (50 μg/mL), with or without isopropyl β-d-1-thiogalactopyranoside (IPTG).

**Table 1:** Strains and plasmids used in this study

Strain/plasmid		Description	Refs.
Strain	WT EPEC	EPEC strain E2348/69, streptomycin resistant	[32]
	EPEC Δ*escN*	Nonpolar deletion of *escN*	[33]
	EPEC Δ*sepD*	Nonpolar deletion of *sepD*	[29]
	Citrobacter rodentium	WT DBS100	[76]
Plasmid	pIFN (pSA10)	50 N-terminal amino acid sequence of EspB fused to human interferon (IFNα2) with YNS mutations	This study
	pT7T318U	Cloned human IFNα2 gene with YNS mutations	[2]
	pHR-CMV-GFP	GFP-expressing lentivirus	[40]

### Construction of the IFN expressing vector

To promote the secretion of IFN_YNS_ via the T3SS, we constructed a vector that expresses a fusion protein consisting of the 50 N-terminal amino acid sequence of EspB and human IFNα2 (with the YNS mutations). To do so, we amplified the *espB* region of EPEC genomic DNA using the EspB_F/EspB_R primer pair (Table 2) and the IFN gene from the vector pT7T318U [2], using the IFN_F/IFN_R primer pair (Table 2). Amplified PCR fragments were fused by PCR to form an espB_50_-IFN fragment. The pSA10 plasmid was amplified using the primer pair pSA10_F/pSA10_R (Table 2). The open plasmid and the fused PCR product were treated with *Dpn*I, purified, and assembled using the Gibson assembly method [34, 35]. The resulting plasmid, termed pIFN, expressed IFN_YNS_ targeted for secretion via the T3SS.

**Table 2:** Sequences of the primers used in this study

Construct/Gene	Primer	Primer sequence	
pIFN in pSA10			
	EspB_F	CAATTTCACACAGGAAACAGATGAATACTATCGATAATAACAATGCGG	
	EspB_R	GACCGGTGGATCCCACAGAAGTTTAGAAATATCCACTCTGCC	
	IFN_F	TGGGATCCACCGGTCATGTGTGATCTGCCGCAG	
	IFN_R	CGGATCCCCGGGAATTCATTCCTTACTTCTTAAACTTTCTTGC	
	pSA10_F	AATTCCCGGGGATCCGTCG	
	pSA10_R	CTGTTTCCTGTGTGAAATTGTTATCCG	
Gene			**Ref.**
*actin*	Actin_F	TCCATCATGAAGTGTGACGT	[50]
	Actin_R	CTCAGGAGGAGGAATGATCT	[50]
*cxcl-10*	CXCL-10_F	CCTGCAAGCCAATTTTGTCCA	This study
	CXCL-10_R	TGTGTGGTCCATCCTTGGAA	This study
*mx2*	MX2_F	TTTTAACCCTCTGGGGACGC	This study
	MX2_R	TAGCGGTCTCACTCTGCTCT	This study
*oas-2*	OAS-2_F	AAGTCAGCTTTGAGCCTCCC	This study
	OAS-2_R	CCAGAACTCAGCTGACCCAG	This study

### In vitro T3S assay

T3S assays were performed as previously described [36–38]. Briefly, EPEC strains were grown overnight in LB, supplemented with the appropriate antibiotics, in a shaker at 37°C. To promote EPEC T3SS expression and assembly, the cultures were diluted 1:20 into pre-heated DMEM supplemented with the appropriate antibiotics and grown statically for 6 h in a tissue culture incubator (with 5% CO_2_) to an optical density of 0.7 at 600 nm (OD_600_). To induce protein expression, 0.25 mM IPTG was added to the bacterial cultures. The cultures were then centrifuged at 20,000 × *g* for 5 min; the bacterial pellets were dissolved in SDS-PAGE sample buffer; and the supernatants that contained the secreted proteins were collected and filtered through a 0.22-μm filter (Millipore). The supernatants were normalized according to the bacterial OD_600_ values and precipitated with 10% (v/v) trichloroacetic acid (TCA) overnight at 4°C to concentrate the proteins. The samples were then centrifuged at 18,000 × *g* for 30 min at 4°C; the precipitates of the secreted proteins were dissolved in SDS-PAGE sample buffer, and the residual TCA was neutralized with saturated Tris. Proteins were analyzed by SDS-PAGE and western blotting.

### Western blot analysis

Samples were subjected to SDS-PAGE and then transferred to nitrocellulose (pore size: 0.45 μm; Amersham Protran) or PVDF (pore size: 0.45 μm; Amersham Hybond) membranes. The blots were blocked for 1 h with 5% (w/v) skim milk-PBST (0.1% Tween in phosphate-buffered saline), incubated with the primary antibody (diluted in 5% skim milk-PBST, for 1 h, at room temperature), washed, and then incubated with the secondary antibody (diluted in 5% skim milk-PBST, for 1 h, at room temperature). Chemiluminescence was detected with Westar Antares ECL reagents (Cyanagen). The following primary antibodies were used: rabbit anti-phosphorylated STAT2 (Abcam Inc.), diluted 1:600; rabbit anti-IFNα2 (Abcam), diluted 1:1000; rabbit anti-JNK1 + JNK2 + JNK3 antibody (Abcam Inc.), diluted 1:1000; mouse anti-DnaK (Abcam, Inc.), diluted 1:5,000, and mouse anti-Tir, which is a generous gift from Prof. B. Brett Finlay (University of British Columbia, Canada). Horseradish peroxidase-conjugated (HRP)-goat anti-mouse and HRP-goat anti-rabbit (Abcam Inc.), diluted 1:10,000, were used as the secondary antibodies. Representative western blots of at least three independent experiments are presented in the Results section.

### Quantification of IFN levels by ELISA

Filtered supernatants of EPEC Δ*sepD* and EPEC Δ*sepD* + pIFN cultures, grown under T3SS-inducing conditions, were analyzed in triplicates by ELISA to determine the IFN_YNS_ concentration using a commercial kit (Human Interferon alpha2 ELISA kit – Abcam Inc.) according to the manufacturer's protocol. Recombinant human IFNα2 was used as a protein standard.

### Antibody neutralization assay

The supernatant sample of EPEC Δ*sepD* + pIFN and a sample of recombinant IFNα (Abcam Inc.), which contain IFNα concentration of 0.5 nM, were left untreated or mixed with the neutralizing anti-human IFNα2 antibody (R&D Systems) at a tenfold excess (5 nM) and incubated for 1 h at room temperature. The samples were then added to Caco-2 cells (at 70% confluence) for 1 h at 37 °C. The cells were then washed and lysed, and their protein extracts were subjected to SDS-PAGE and western blot analysis using antibodies against phosphorylated STAT2 and actin (loading control). A recombinant IFNβ (0.5 nM) or human IFNα2 (0.5 nM) were used as positive controls, while untreated cells and cells incubated with supernatant from a culture of EPEC Δ*sepD* were used as negative controls.

### RNA extraction and cDNA preparation

HeLa cells were incubated for 8 h with the supernatants of EPEC Δ*sepD* or EPEC Δ*sepD* that expresses IFN (Δ*sepD* + pIFN). Untreated HeLa cells and HeLa cells incubated with commercial IFNβ (2 nM; Pepro-Tech) were used as negative and positive controls, respectively. Following incubation, 1 × 10^6^ cells were collected and subjected to RNA extraction using the TRIzol reagent according to the manufacturer's guidelines (Invitrogen). Total RNA was resuspended in 30 μL of diethyl-pyro-carbonate (DEPC)-treated RNase-free water, and its quality was assessed by agarose gel electrophoresis. One microgram of RNA was taken from each sample for cDNA synthesis using Protoscript II First Strand cDNA Synthesis Kit (NEB) with the oligo (dT)18 primer according to the manufacturer’s protocol. Samples were stored at -20°C.

### Quantitative PCR (qPCR) analysis

Transcript-specific PCR primer pairs were designed using the primer BLAST tool (NCBI). Forward and reverse primers were chosen in different exons to minimize noise from DNA contamination, and melting curve analysis was used to ensure the specificity of each primer pair. The sequences of the primers are presented in Table 2. RT-qPCR reactions with the cDNA of the examined samples, gene-specific primers, and SYBR Green I mix (Roche) were analyzed in triplicate in a QuantStudio cycler (Applied Biotechnologies, Thermo). A standard curve was constructed in each experiment by using fivefold serial dilutions of the purified template. The reaction conditions for amplification were: initial denaturation at 95 °C for 2 min and 35 cycles of 95 °C for 20 s, cooling to 60 °C for 20 s, followed by 72 °C for 20 s while monitoring fluorescence. Post-amplification melting-curve analyses were performed to confirm reaction specificity. The expression levels of the target genes of the different treatments were normalized to the actin housekeeping gene and compared using a relative quantification method [39]. Real-time data are presented as the fold change in expression levels.

### Anti-proliferation assay

HeLa cells (2.5 × 10^3^ cells per well) were grown overnight in flat-bottomed microtiter plates and then incubated with serial dilutions of the extracted supernatants. The supernatants were prepared as described above in the “In vitro T3S assay” section without antibiotic supplementation of the bacterial growth medium. The TCA-precipitated proteins were resuspended in 1 mL of DMEM. After the addition of the supernatant extracts (or commercial IFNβ as the control), antiproliferation activity was monitored after 96 h. Cell viability was determined by crystal violet staining, as described previously [40], or by MTT assay [41].

### Antiviral assay

For evaluating antiviral activity induced by IFN_YNS_, 1.5 × 10^4^ HeLa cells were grown overnight in a 24-well plate. Cells were then incubated for 4 h with serial dilutions of supernatant extracts collected from either EPEC Δ*sepD* or EPEC Δ*sepD* + pIFN. Thereafter, the cells were transduced with a GFP-expressing lentivirus (VSV-G pseudotyped lentivirus with a pHR-CMV-GFP vector) at an MOI of 1 [42]. Cells were harvested 48 h post-transduction and subjected to FACS analysis of the GFP signal. The percentage of GFP-expressing cells in the treated samples was calculated relative to the number of GFP-expressing cells in the untreated HeLa sample. Commercial IFNβ was used as a positive control.

### Statistical analysis

For statistical analysis, the IBM SPSS Statistics 27.0 package was used. An independent 2-tailed t-test with assumed equal variances was performed for anti-proliferation and anti-viral assays. To evaluate differences in gene induction levels detected by RT-PCR, ANOVA tests with post-hoc testing for multiple comparisons were used. STD was used for error bars.

### Translocation assays

Translocation assays were performed as previously described [43]. Briefly, HeLa cells (8 × 10^5^ cells per well) were infected for 3 h with EPEC strains that had been pre-induced for 3 h for T3SS activity (pre-heated DMEM, statically, in a CO_2_ tissue culture incubator). Cells were then washed with cold PBS, collected, and lysed with RIPA buffer. Thereafter, samples were centrifuged at 18,000 × *g* for 5 min to remove non-lysed cells, and supernatants were collected, mixed with SDS-PAGE sample buffer, and subjected to western blot analysis with anti-JNK and anti-actin (loading control) antibodies. Untreated samples, samples infected with non-transformed EPEC strains, and a sample infected with the Δ*escN* mutant strain transformed with pIFN were used as negative controls.

## Results

### EPEC can secrete human IFN_YNS_ through the T3SS

**Fig. 1:**
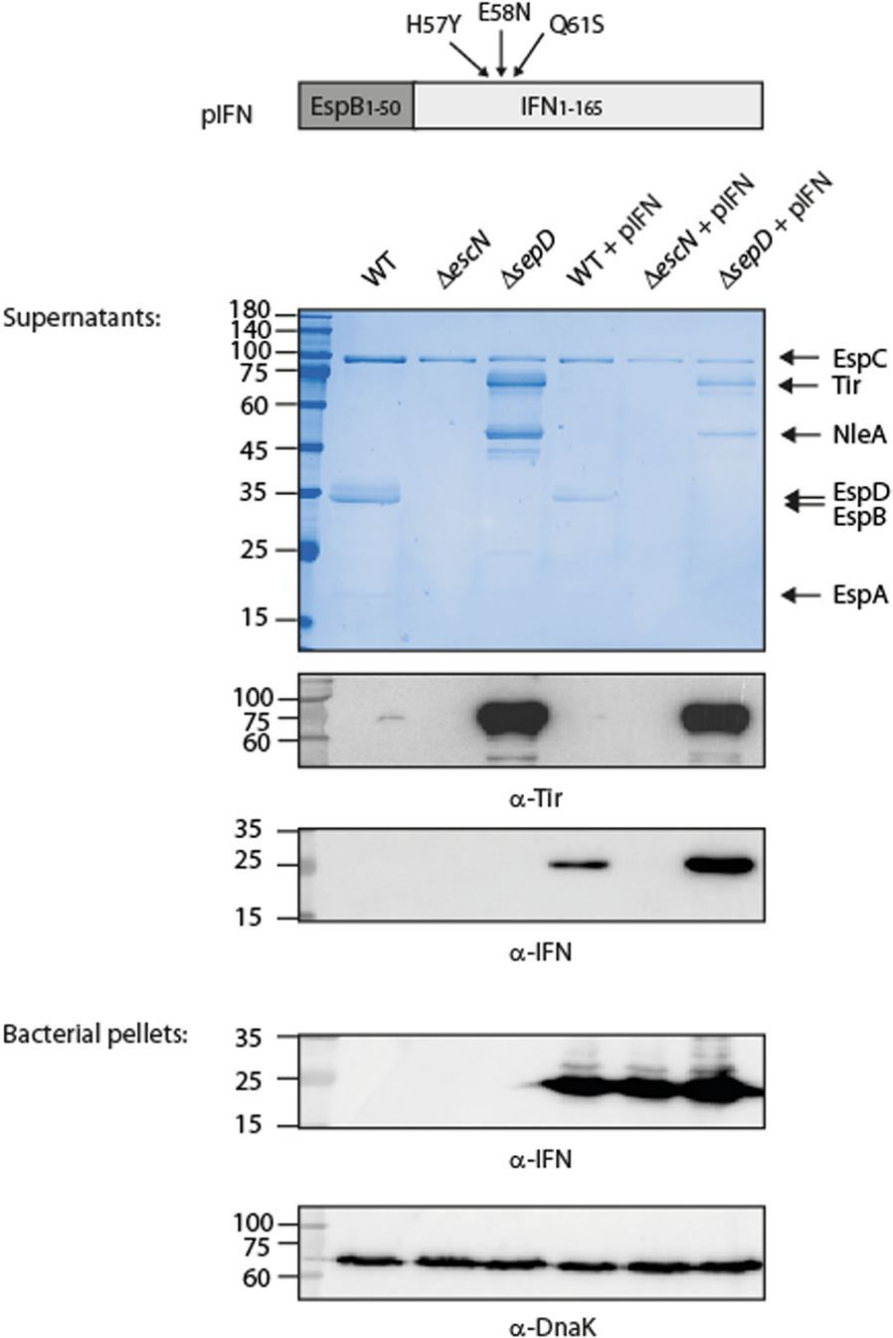
IFN_YNS_ can be secreted by the T3SS of EPEC. WT EPEC, Δ*escN* (a T3SS ATPase mutant), and Δ*sepD* (a hypersecreting mutant) strains and WT, Δ*escN*, and Δ*sepD* strains that express IFN_YNS_ (+ pIFN) were grown under T3SS-inducing conditions, and their supernatants and pellets were separated and normalized according to bacterial OD_600_ values. The proteins from the secreted fractions were concentrated from the supernatants of bacterial cultures and analyzed by SDS-PAGE and Coomassie staining or western blot with anti-IFNα2 and anti-Tir antibodies. The bacterial pellets were analyzed by SDS-PAGE and western blot with anti-IFNα2 and anti-DnaK antibodies

To promote IFN_YNS_ secretion via the T3SS, we fused the sequence of the first 50 residues of EspB, a T3SS translocator protein, to the N-terminal of the IFN_YNS_ protein (Fig. 1). This short EspB sequence can efficiently direct fused proteins to T3SS-dependent secretion [28]. To investigate whether the fused IFN_YNS_ (25 kDa) is secreted through the T3SS, we transformed the IFN_YNS_ expression vector, pIFN, into wild-type (WT) EPEC and the Δ*escN* and Δ*sepD* (see below) null strains. The strains were cultured under T3SS-inducing conditions and then centrifuged to separate between the bacterial pellets (expression) and their supernatants (secretion). The samples were subjected to SDS-PAGE followed by Coomassie staining or western blot analysis using anti-IFN, anti-Tir, and anti-DnaK antibodies. IFN_YNS_ was detected in the bacterial pellet and the supernatant samples of WT EPEC expressing IFN_YNS_ (Fig. 1). To confirm that this secretion of IFN_YNS_ was indeed dependent on the T3SS, we examined the secretion of IFN_YNS_ in the Δ*escN* mutant strain, which has a nonfunctional T3SS complex due to deletion of the T3SS ATPase gene [33]. Our results show that IFN_YNS_ was detected in the pellet of the Δ*escN* mutant strain but not in the secreted supernatant fraction (Fig. 1), thus indicating that the secretion of IFN_YNS_ was dependent on the T3SS. Additionally, we found that the secretion of IFN_YNS_ was enhanced in the Δ*sepD* strain, as expected. This strain is characterized by an upregulated release of T3SS effectors, as shown in the Coomassie staining and anti-Tir blot (Fig. 1), resulting from the deletion of a substrate secretion regulator [20, 28]. Overall, our results indicate that human IFN_YNS_ can be produced by EPEC and secreted into the growth medium under T3SS-inducing conditions.

### Bacterially produced IFN is biologically active

**Fig. 2:**
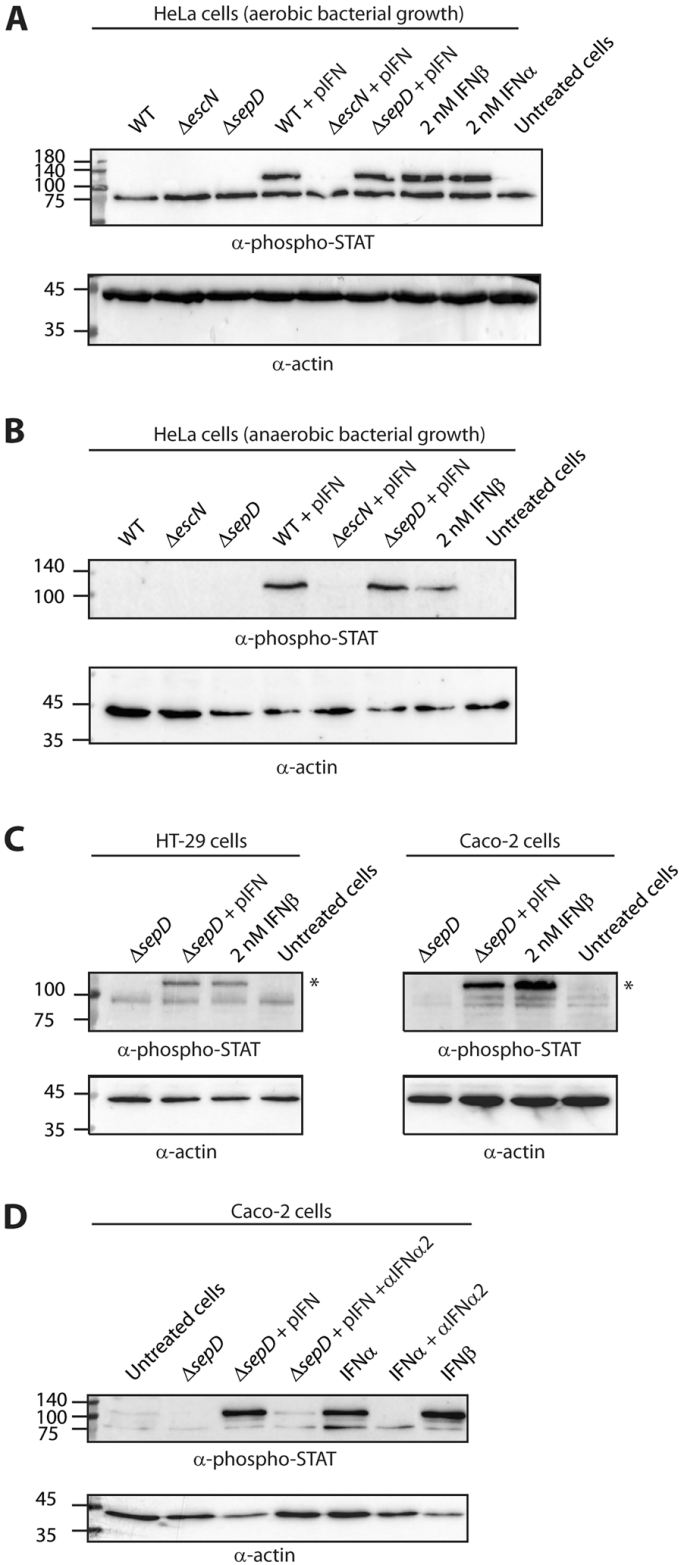
Bacterially secreted IFN_YNS_ induces activation of the IFN-1 pathway. Cells were incubated with supernatants of bacterial cultures, washed, lysed, and their protein extracts were subjected to SDS-PAGE and western blot analysis using antibodies against phosphorylated STAT2 (phospho-STAT) and actin (loading control). Cells incubated with commercial IFNβ or IFNα (2 nM) were used as positive controls, while a sample of untreated cells was used as a negative control. **A** HeLa cells were incubated with supernatants collected from cultures of WT EPEC, Δ*escN*, and Δ*sepD* strains in the presence or absence of a plasmid encoding for IFN_YNS_ (pIFN) that were grown aerobically or **B** anaerobically. **C** HT-29 (left) and Caco-2 (right) cells were incubated with supernatants from cultures of EPEC Δ*sepD* and EPEC Δ*sepD* that express and secrete IFN_YNS_ (pIFN). **D** Caco-2 cells were incubated with supernatants from a culture of EPEC Δ*sepD* + pIFN and with commercial IFNα2 (0.5 nM), alone or following pre-incubation with an anti-IFNα2 antibody (5 nM) for 1 h. IFNβ (0.5 nM) was used as a positive control, and untreated cells and cells incubated with supernatant from a culture of EPEC Δ*sepD* were used as negative controls

To examine whether EPEC-secreted IFN is biologically active, we studied the ability of EPEC strains secreting IFN to activate IFN signaling pathways. Since it is known that the binding of IFN to its receptor (IFNAR) initiates an intracellular response that leads to the phosphorylation of the cellular STAT2 protein [1], we followed the levels of phosphorylated STAT2 in HeLa cells grown in media supplemented with filtered supernatants from EPEC cultures grown under T3SS-inducing conditions (to promote IFN secretion through the T3SS). The addition of the supernatant of WT EPEC that expresses IFN_YNS_ (WT + pIFN) led to phosphorylation of STAT2, with the levels being similar to those obtained with commercial IFNβ or IFNα (2 nM) (Fig. 2A). These results indicate that EPEC-secreted IFN is biologically active. To exclude the possibility that the activation of the IFN pathway was due to bacterial components in the supernatants and not specifically to the secreted IFN, we added the supernatants of EPEC strains that do not express IFN_YNS_ (WT, Δ*escN*, and Δ*sepD*) to HeLa cells and did not detect phosphorylated STAT2 in these samples. We, therefore, concluded that the activation of the IFN pathway by the WT + pIFN supernatant was explicitly induced by the presence of IFN_YNS_ (Fig. 2A). We also observed that STAT2 phosphorylation in HeLa cells incubated with the supernatant of the hyper-secreteing strain Δ*sepD* + pIFN culture was similar to that of cells incubated with the supernatant of WT EPEC expressing IFN_YNS_ (WT + pIFN) and with commercial IFNβ or IFNα. However, the addition of the supernatant of T3SS-deficient mutant strain, Δ*escN* EPEC, that expresses IFN (Δ*escN* + pIFN) but does not secrete it, resulted in only a minimal level of phosphorylated STAT2 (Fig. 2A). These results confirm that the IFN pathway is primarily activated by T3SS-secreted IFN and not by IFN released from lysed IFN-expressing bacteria.

To investigate whether IFN_YNS_ secretion would occur under conditions that better simulate the gut environment, we grew the bacteria anaerobically and then added the bacterial supernatants to HeLa cells. We observed that only the WT + pIFN supernatant and the Δ*sepD* + pIFN supernatant triggered phosphorylation of STAT2, similar to commercial IFNβ (2 nM) (Fig. 2B). In contrast, there was no phosphorylated STAT2 signal in samples supplemented with the supernatants of Δ*escN* + pIFN or of bacterial strains that do not express IFN (Fig. 2B). Following our observation that IFN secreted from EPEC grown under aerobic/anaerobic conditions can activate the IFN pathway in HeLa cells, we expended our examination to cell lines derived from human epithelial cells originated in large intestine and colon. Given that the Δ*sepD* + pIFN strain exhibits hyper-secretion of IFN through the T3SS, coupled with its reduced ability to infect host cells, we prioritized the investigation of this strain to explore the ability of bacterial-secreted IFN to induce an IFN response. Bacterial supernatants were added to HT-29 and Caco-2 cells to follow the phosphorylated STAT2 signal. We observed that the Δ*sepD* + pIFN supernatant triggered phosphorylation of STAT2, similarly to commercial IFNβ (2 nM), while no phosphorylation was observed with the supernatant of EPEC Δ*sepD* (Fig. 2C). To validate that the activation of the STAT pathway was explicitly induced by the IFN secreted to the extracellular medium of EPEC cultures, we examined the ability of anti-IFNα2 antibody to neutralize the effect of IFN found in the supernatant of EPEC Δ*sepD* + pIFN culture. Using the human Interferon alpha 2 ELISA kit, we determined the concentration of IFN in the bacterial supernatant to be approximately 0.5 nM. Subsequently, recombinant IFNα2 and IFNβ were introduced at a similar concentration. As expected, we observed that addition of recombinant IFNα2 and IFNβ (0.5 nM) induces robust STAT2 phosphorylation, while very low levels of phosphorylated STAT2 were observed in untreated cells or cells incubated with the supernatant of EPEC Δ*sepD* culture (Fig. 2D). Treatment with the supernatant of EPEC Δ*sepD* + pIFN culture triggered a robust STAT2 phosphorylation in Caco-2 cells, which was substantially reduced when the supernatant was pre-incubated with the anti-IFNα2 antibody (Fig. 2D). A similar neutralizing effect was observed for recombinant IFNα2 that was preincubated with anti-IFNα2 antibody (Fig. 2D). These findings suggest that EPEC can efficiently secrete biologically active IFN in anaerobic gut-simulating conditions, eliciting a subsequent response in gut-derived cells. Consequently, it implies that bacteria secreting IFN are likely to generate biologically active IFN when orally administered and cultivated in vivo.

### EPEC-secreted IFN upregulates ISG transcription

**Fig. 3:**
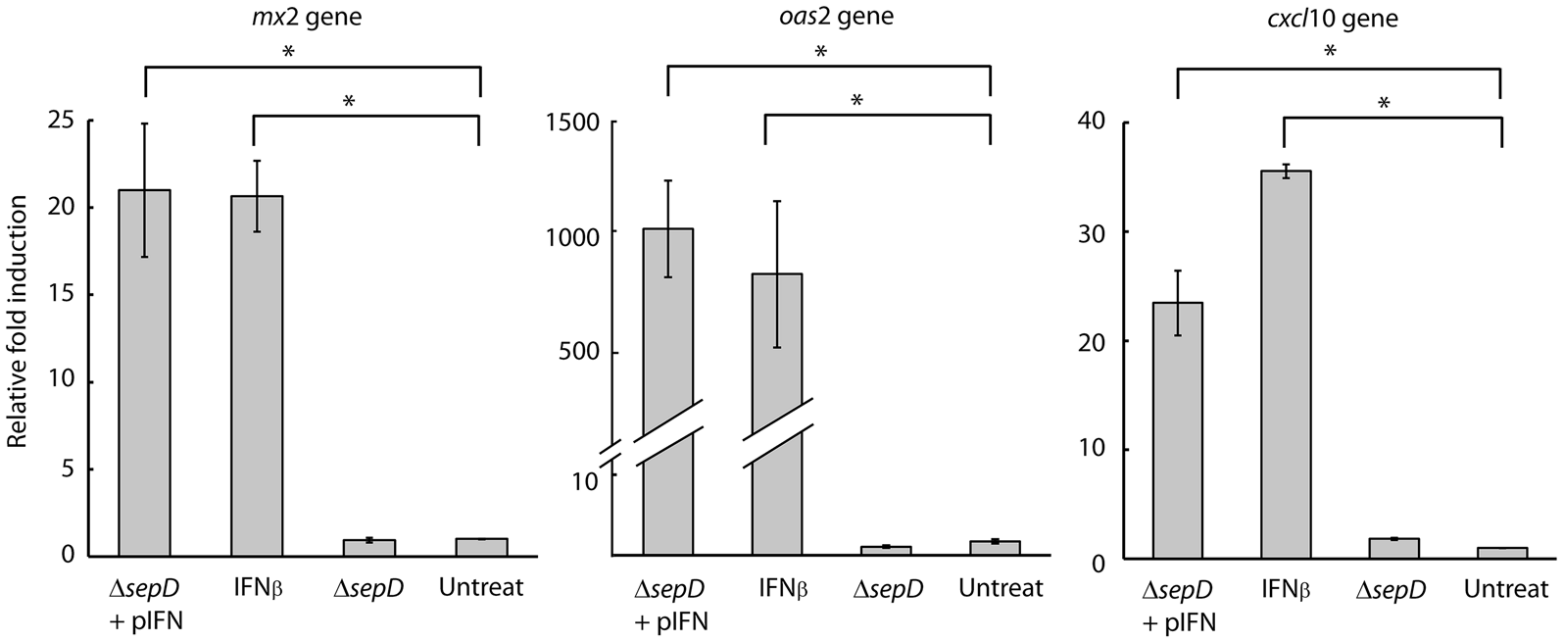
Bacterially secreted IFN_YNS_ upregulates interferon-stimulated genes. HeLa cells were incubated with bacterial supernatants of Δ*sepD* EPEC, Δ*sepD* expressing IFN_YNS_ (Δ*sepD* + pIFN), or commercial IFNβ. Transcription levels of three interferon-stimulated genes (*mx*2, *oas*2, and *cxcl*10) were determined by qRT-PCR and are presented as fold induction relative to untreated cells. A representative experiment (n = 3) is presented. Bars represent the standard error, *P < 0.05

To evaluate the effect of EPEC-secreted IFN on the regulation of ISGs, we examined the changes at the transcriptional levels of two “antiviral” ISGs, namely *oas*2 and *mx*2, and one "immunomodulatory and antiproliferation" ISG, namely, *cxcl*10. We, therefore, incubated HeLa cells with purified supernatants from either Δ*sepD* EPEC alone (Δ*sepD*) or Δ*sepD* expressing IFN (Δ*sepD* + pIFN). Samples of untreated HeLa cells or HeLa cells treated with commercial IFNβ (2 nM) served as negative and positive controls, respectively. Our results showed that exposure of HeLa cells to EPEC-secreted IFN induced a significant upregulation of ISG transcription, similar to the upregulation induced by commercial IFNβ (Fig. 3). In contrast, incubation of HeLa cells with the supernatant of Δ*sepD* EPEC did not induce upregulation of ISG transcription and resulted in a similar transcription level to that of the untreated control (Fig. 3). These results confirm that the ISG upregulation observed in HeLa cells incubated with the Δ*sepD* + pIFN supernatant was specific to the ability of the strain to express and secrete IFN and did not result from a cellular response to general bacterial components in the supernatants.

### EPEC-secreted IFN_YNS_ induces antiproliferation of HeLa cells in vitro

**Fig. 4:**
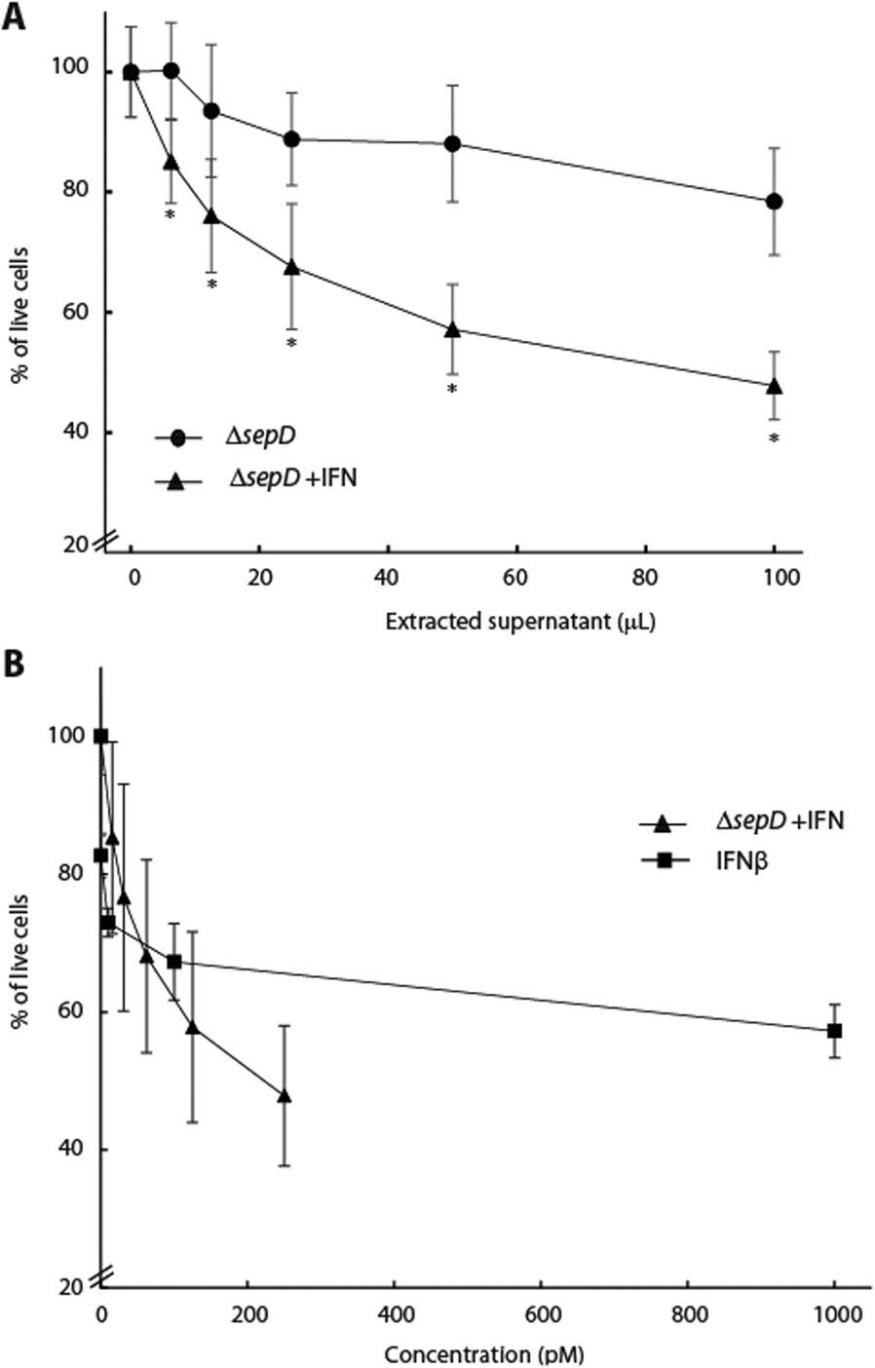
Bacterially secreted IFN_YNS_ shows antiproliferation activity. HeLa cells were incubated with extracts of bacterial supernatants of either Δ*sepD* EPEC or Δ*sepD* expressing IFN_YNS_ (Δ*sepD* + pIFN) and their viability after 96 h is presented as a function of the volume of the bacterial extracts (**A**) or IFN concentration (**B**). The antiproliferation activity of recombinant IFNβ is also depicted in (**B**), serving as a comparative reference for the activity of bacteria-secreted IFN_YNS_. Bars represent the standard deviation; *P < 0.05

Type I IFNs inhibit cell proliferation and are therefore used in treating human malignancies [44–51]. To evaluate the effect of EPEC-secreted IFN_YNS_ on cell proliferation, we used HeLa cells, which multiply rapidly and were previously reported to activate apoptotic response following IFNα treatment [50]. For that purpose, cells were incubated with various concentrations of purified supernatants collected from either Δ*sepD* EPEC or Δ*sepD* EPEC expressing IFN (Δ*sepD* + pIFN) for 96 h and cell viability was quantified by crystal violet staining. We observed that incubation of HeLa cells with EPEC-secreted IFN_YNS_ significantly inhibited cell growth in a dose-dependent manner (Fig. 4A). The addition of the maximal volume (100 μL/well; of ~ 250 pM concentration) of the supernatant of Δ*sepD* + pIFN resulted in a dramatic and statistically significant reduction in cell viability (~ 50%). In contrast, the addition of the same volume of Δ*sepD* supernatant induced a much milder reduction in cell viability (20%) that probably resulted from small amounts of bacterial components, such as lipopolysaccharides, which are found in the supernatant (Fig. 4A). Similar results were observed when HeLa cells viability was evaluated, using the MTT method (data not shown). To assess the results in comparison to commercial IFNβ, we plotted the percentage of live cells as a function of IFN concentration found in the bacterial culture or the recombinant IFNβ (Fig. 4B). These results suggest that bacteria-secreted IFN_YNS_ can trigger a similar antiproliferation response as recombinant IFNβ.

### EPEC-secreted IFN_YNS_ exhibits an antiviral effect in vitro

**Fig. 5:**
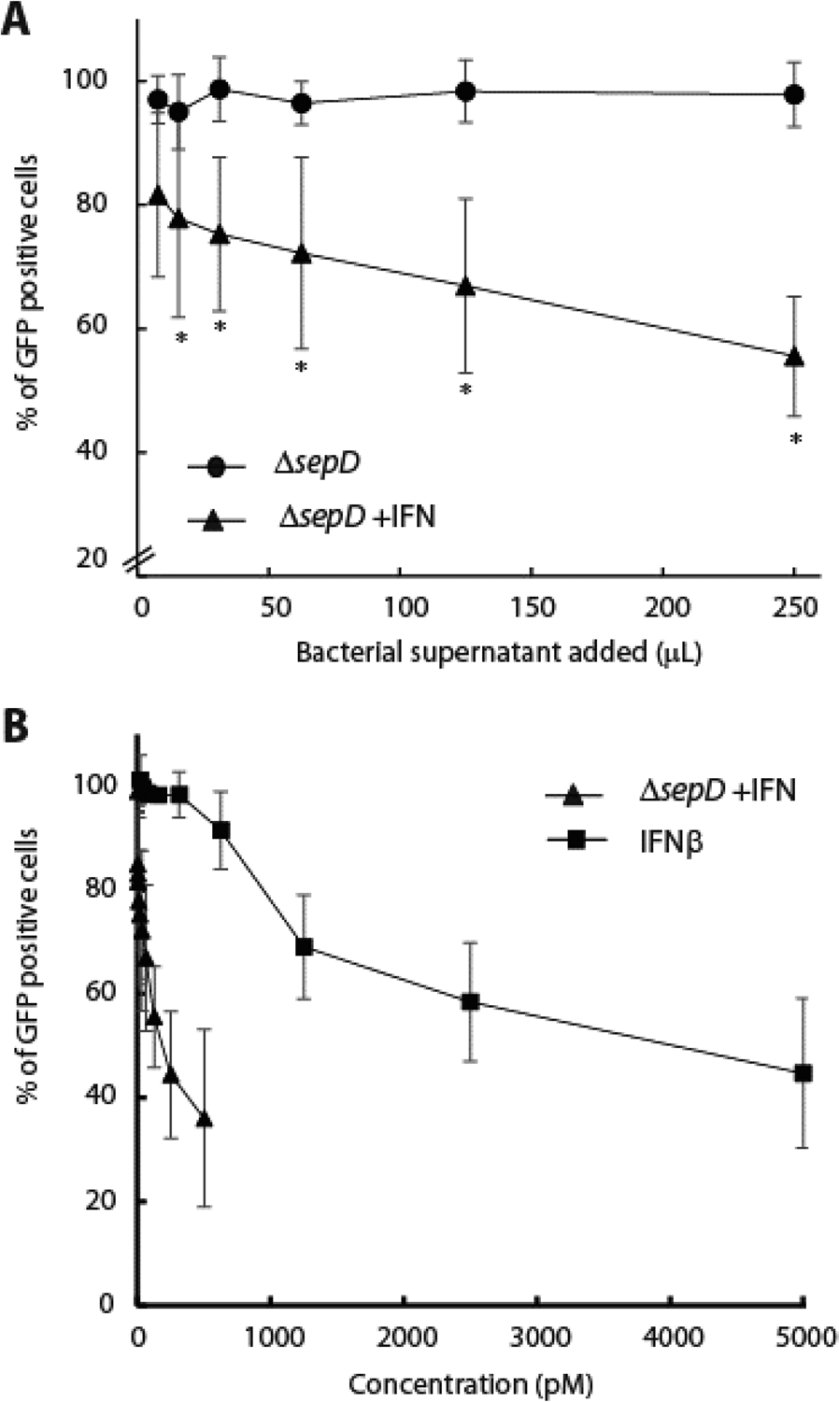
Bacterially secreted IFN_YNS_ shows antiviral activity. HeLa cells were incubated with extracts of bacterial supernatants of either Δ*sepD* EPEC or Δ*sepD* expressing IFN_YNS_ (Δ*sepD* + pIFN) for 4 h before being transduced with a GFP-expressing pseudovirus at an MOI of 1. Cells were harvested 48 h post-transduction and subjected to FACS analysis for monitoring GFP expression. The results are presented as a percentage of GFP-positive cells relative to GFP-positive cells of the untreated control sample, which was not pre-incubated with bacterial supernatant. The antiviral activity is presented as a function of the volume of the bacterial extracts (**A**) or IFN concentration (**B**). The antiviral activity of recombinant IFNβ is also depicted in (**B**), serving as a comparative reference for the activity of bacteria-secreted IFN_YNS_. Bars represent the standard deviation; *P < 0.05

The induction of the IFN response is one of the first lines of defense against viral infection. Our transcription analysis reflected this antiviral response in the upregulation of the antiviral genes, *oas*2 and *mx*2 (Fig. 3). To examine whether bacterially produced IFN_YNS_ could enhance the antiviral response in vitro, we used HeLa cells infected with a GFP-expressing pseudovirus as a model. Engineered viruses of this type are VSV-G pseudotyped viruses that can efficiently transduce target cells, but cannot produce progeny particles, allowing only a single infection cycle. HeLa cells were pre-treated with supernatants of either Δ*sepD* + pIFN or Δ*sepD* cultures for 4 h prior to the viral transduction at a multiplicity of infection (MOI) of 1 [42]. Cells were then washed and incubated with the pseudovirus for 48 h, harvested, and subjected to FACS analysis to determine GFP expression as a measure of viral infection. The percentage of GFP-expressing cells of the pre-treated samples relative to the level of GFP-expressing cells of an untreated sample that was infected with the lentivirus is shown in Fig. 5. We found that incubation of HeLa cells with the supernatant of a Δ*sepD* + pIFN culture before viral infection reduced viral entry into the cells in a dose-dependent manner (Fig. 5A). The most pronounced effect was observed for the HeLa cell culture that was supplemented with the maximal volume of Δ*sepD* + pIFN supernatant, namely, where we observed a 50% reduction of lentiviral expression compared to cells that were incubated with similar volume of control Δ*sepD* supernatant (Fig. 5A). Representative immunofluorescence images demonstrated that uninfected cells lack GFP signal, whereas cells infected with viral particles without pre-treatment exhibit a high number of GFP-positive cells (Additional file 1: Fig. S1). Furthermore, the viral infection of cells incubated with the Δ*sepD* supernatant resulted in a comparable number of GFP-positive cells as the untreated cells, whereas cells exposed to Δ*sepD* + pIFN supernatant show a noticeable reduction in the number of GFP-positive cells (Additional file 1: Fig. S1). To ensure that the antiviral response was unaffected by the antiproliferation response, we specifically measured the GFP signal in viable cells (Additional file 1: Fig. S2). Additionally, given the rapid nature of the IFN-activated antiviral response, the cells were exposed to IFN for only a short period (4 h) before the viral infection. Such exposure was not expected to trigger the slower IFN-induced antiproliferation response (measured after 96 h of incubation with the supernatants). These results confirmed that EPEC-secreted IFN_YNS_ could indeed promote an antiviral response. To assess the results in comparison to commercial IFNβ, we plotted our results as a function of IFN concentration found in the bacterial culture along with the percentage of GFP-positive cells after treatment with recombinant IFNβ (Fig. 5B). These results suggest that the bacteria-secreted IFN_YNS_ trigger an enhanced antiviral response compared to the IFNβ. To investigate whether additional bacterial components, apart from IFN_YNS_, are released from the Δ*sepD* + pIFN strain to induce a synergistic antiviral effect, we assessed the antiviral effect of IFNβ alone and when combined with the supernatant sample of Δ*sepD*. Our finding revealed that IFNβ triggered a comparable antiviral response regardless of the medium in which it was introduced (Additional file 1: Fig. S3). We, therefore, hypothesize that the higher antiviral response we observed for IFN_YNS_, a mutant version of IFNα2, compared to IFNβ may stem from either its enhanced affinity to the IFNAR found on HeLa cells or its ability to elicit a more potent response against the lentivirus employed in our experimental system [52, 53].

### IFN_YNS_-secreting EPEC bacteria can directly activate the IFN signaling pathway

**Fig. 6:**
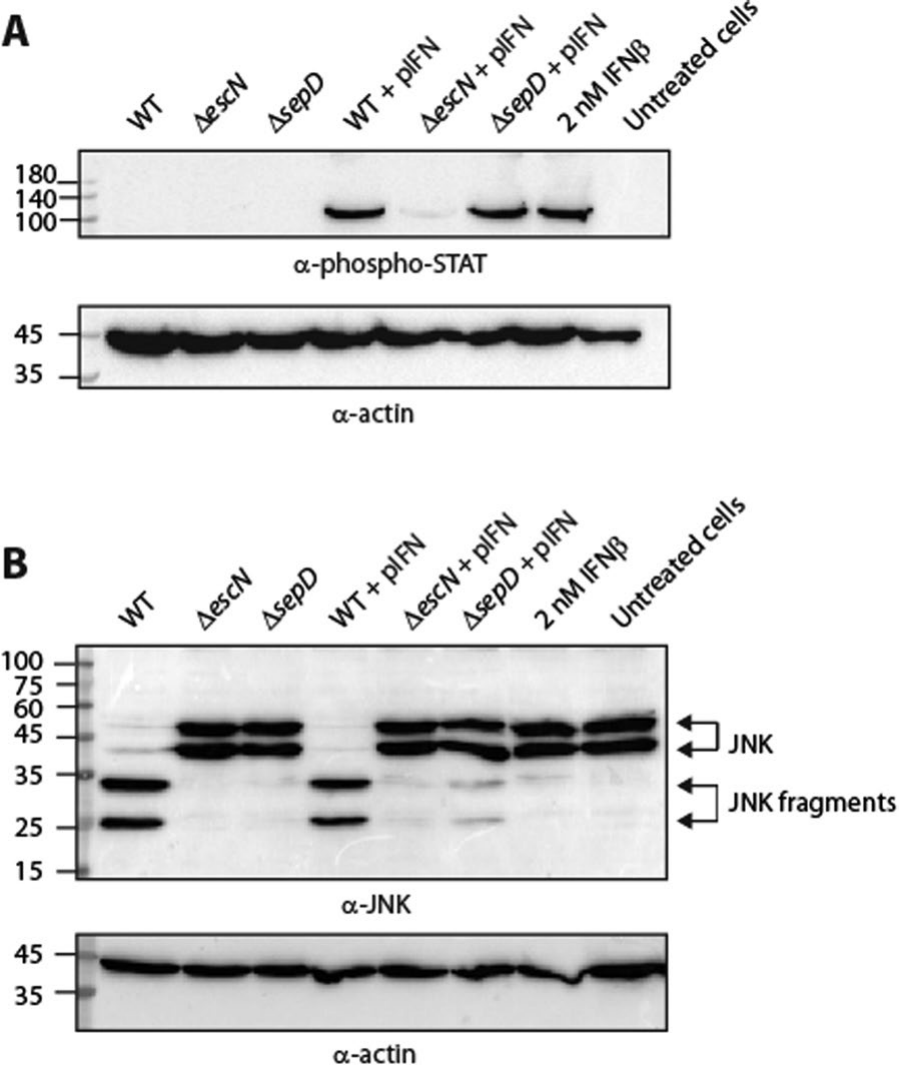
The addition of IFN_YNS_-secreting bacteria to host cells induces IFN-1 pathway activation. WT EPEC, Δ*escN*, and Δ*sepD* strains in the presence or absence of a plasmid-encoding human IFN (pIFN) were added to the HeLa cells and co-cultured for 3 h. The cells were then washed and lysed, and their protein extracts were subjected to SDS-PAGE and western blot analysis using anti-phosphorylated STAT2 (phospho-STAT) (**A**) or anti-JNK (**B**) and anti-actin antibodies (loading control). JNK and its degradation fragments are indicated on the right of the gel

To examine whether incubation with secreting bacteria, and not the purified supernatant, could directly induce activation of the IFN signaling pathway, we incubated various EPEC strains directly with HeLa cells. The cells were then washed and lysed, and samples were subjected to western blot analyses using anti-phosphorylated STAT2 and anti-actin antibodies. Our results showed that incubation of HeLa cells with EPEC strains that actively secrete IFN_YNS_ (i.e., WT + pIFN and Δ*sepD* + pIFN) enhanced STAT2 phosphorylation, similar to the activation with commercial IFNβ (Fig. 6A). In contrast, incubation of HeLa cells with the EPEC Δ*escN* + pIFN strain (not secreting IFN_YNS_) showed minimal phosphorylation of STAT2 (Fig. 6A), and HeLa cells incubated with EPEC strains that had not been manipulated to express IFN_YNS_ (WT, Δ*escN*, or Δ*sepD*) did not produce a phosphorylated-STAT2 signal (Fig. 6A). Overall, these results suggest that the IFN pathway was activated in the HeLa cells due to the active secretion of IFN_YNS_ into the extracellular environment and not due to exposure of the cells to general bacterial components. Since WT EPEC is virulent and infects host cells, it cannot be used to deliver IFN_YNS_ orally. However, EPEC Δ*sepD* is expected to be an attenuated strain, as its substrate regulation is defective (it does not secrete essential components needed for host infection), and it may, therefore, be used as a safe vehicle for oral IFN_YNS_ delivery. To confirm this premise, we examined the infectivity of various EPEC strains (WT, Δ*escN*, Δ*sepD*, WT + pIFN, Δ*escN* + pIFN, and Δ*sepD* + pIFN) by assessing their ability to translocate effectors into host cells. For this purpose, we infected HeLa cells with bacteria, washed them, and collected cell samples. The samples were examined for the cleavage pattern of JNK, a host protein that is cleaved by a translocated EPEC effector known as NleD [43]. As expected, regardless of IFN_YNS_ expression, WT EPEC induced extensive degradation of JNK relative to the untreated sample and the sample infected with the Δ*escN* mutant strain (Fig. 6B). Importantly, infection of HeLa cells with Δ*sepD* and Δ*sepD* + pIFN caused very mild degradation of JNK, relative to WT EPEC (Fig. 6B), thus confirming that the Δ*sepD* EPEC strain has a significantly reduced ability to infect host cells. These results are in keeping with a previous report showing that the corresponding deletion of the *sepD* gene in the EPEC-related mouse pathogen, *C. rodentium*, is non-virulent in mice [31].

**Fig. 7:**
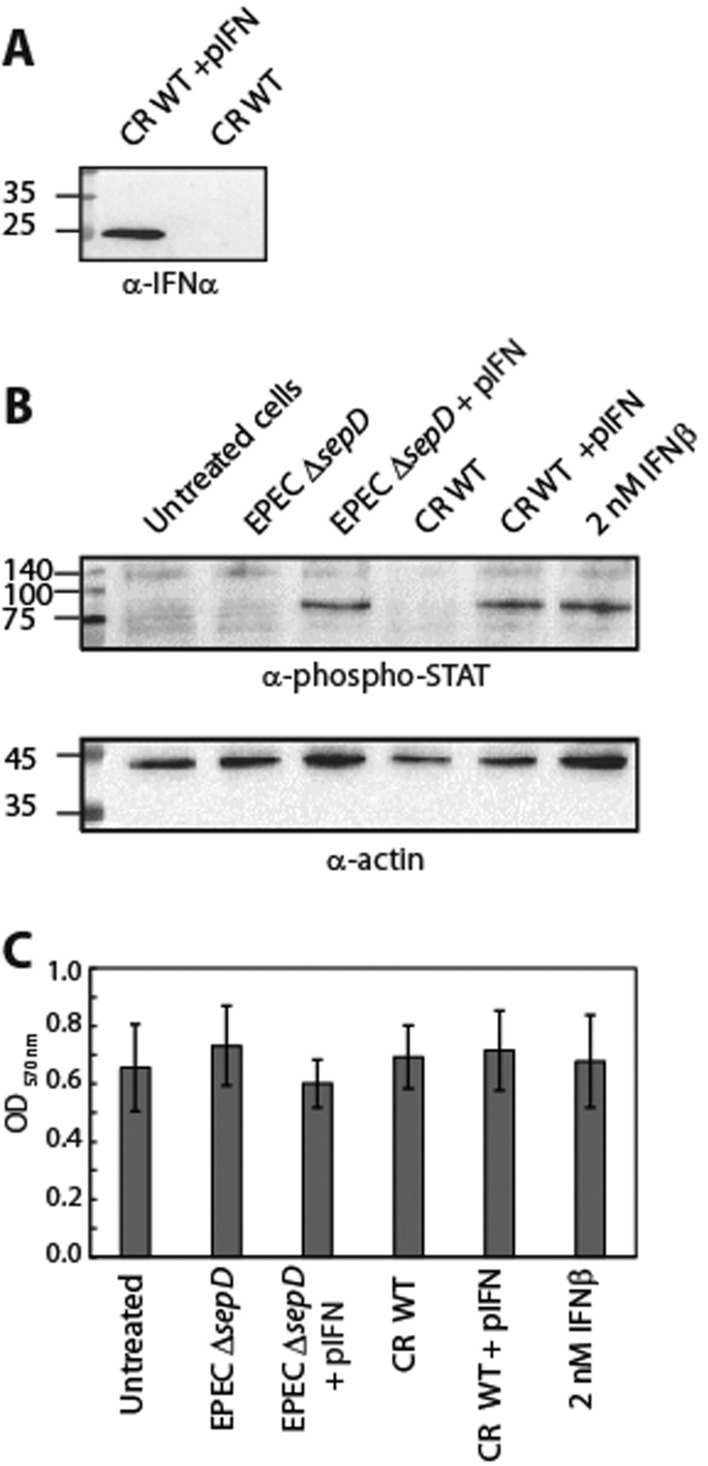
IFN_YNS_ secreted from *C. rodentium* induces IFN-1 pathway activation. **A** Cultures of *C. rodentium* (CR) in the presence or absence of IFN_YNS_ encoding plasmid (+ pIFN) were grown under T3SS-inducing conditions, and their supernatants and pellets were separated and normalized according to bacterial OD_600_ values. The secreted fractions were concentrated from the supernatants of the bacterial cultures and analyzed by SDS-PAGE and western blot with an anti-IFNα2 antibody. **B** HeLa cells were incubated with supernatants from cultures of EPEC Δ*sepD*, EPEC Δ*sepD* that express and secrete IFN_YNS_ (pIFN), *C*. *rodentium*, and *C. rodentium* that express and secrete IFN_YNS_ (pIFN). Commercial-available IFNβ (2 nM) and untreated cells were used as positive and negative controls, respectively. **C** HeLa cells incubated with EPEC or *C. rodentium* bacterial supernatants were assessed for cell viability using MTT assay. Mean OD_570 nm_ values are presented

To examine whether a non-human pathogen can induce a similar IFN response as EPEC, we transformed the IFN_YNS_ expression vector into *C. rodentium*, the natural murine intestinal pathogen, which is considered non-pathogenic to humans [54]. Cultures of *C. rodentium* in the presence or absence of IFN_YNS_ (+ pIFN) were grown under T3SS-inducing conditions, and their supernatants and pellets were separated. The secreted fractions were concentrated from the supernatants of the bacterial cultures and analyzed by SDS-PAGE and western blot with an anti-IFNα2 antibody. IFNα was detected in the supernatant sample of *C. rodentium* expressing IFNα but not in the wildtype *C. rodentium* strain (Fig. 7A). To confirm that the IFNα secreted from *C. rodentium* is functional, we incubated HeLa cells with filtered supernatants from cultures of EPEC and *C*. *rodentium* that express and secrete IFN_YNS_ (pIFN). We observed that the addition of supernatants of EPEC and *C*. *rodentium* cultures that express IFN_YNS_ showed a significant phospho-STAT2 signal, comparable to the one obtained by commercial IFNβ (2 nM) (Fig. 7B). Incubation of the supernatants of the parental strains (EPEC Δ*sepD* and *C*. *rodentium*) with HeLa cells did not result in phosphorylated-STAT (Fig. 7B). To ensure that HeLa cell viability was not significantly impacted by incubation with supernatants from EPEC and *C*. *rodentium*, we evaluated cell viability at the end of the incubation period using the MTT assay. Our results revealed comparable OD_570 nm_ values across all treatments, suggesting that the addition of supernatants from both EPEC and *C*. *rodentium* did not alter cell viability (Fig. 7C). Overall, these results demonstrate that the ability to secrete IFN via the T3SS is not species-specific and can be easily adjusted to other related bacteria. We showed that IFNα secreted from *C*. *rodentium* induces a similar IFN response as IFNα secreted from EPEC. Since *C*. *rodentium* is considered a non-human pathogen, it might provide a safer way to deliver IFNα in future clinical studies.

## Discussion

Many proteins are attractive therapeutic molecules, but their susceptibility to degradation in the digestive system precludes their oral administration; therefore, they are currently given parenterally [55–57]. In the past few decades, significant research has thus been invested in developing effective oral delivery strategies for protein-based drugs, such as microencapsulation in various polymers or vesicles. While this research has produced encouraging results, many challenges remain, including protein stability during production, long-term storage, regulated in vivo release, and the cost of producing encapsulated drugs.

For various applications, exploitation of the bacterial T3SS has been suggested as a potential tool for the secretion and delivery of proteins, including reporter proteins, enzymes, and antigens for vaccine development [27, 58–62]. However, since the secreted proteins must unfold during their translocation through the T3SS conduit, it is critical to determine, for each therapeutic protein, its ability to refold in the extracellular space and regain its biological activity [63]. Furthermore, while the potential of this system is thought to be enormous, only a few studies have actually employed it to deliver potentially therapeutic proteins [58, 64, 65]. In this study, we present promising results that IFN, which belongs to a large family of immune-modulatory proteins, can be secreted by the T3SS of EPEC while remaining functional, opening up new avenues for oral delivery of IFN by secreting bacteria. We believe that this system will have many advantages as an oral administration platform, including cheap and efficient production, protein stability, release at desired sites (the T3SS is activated after reaching the small intestine), a transient effect that will be better controlled (by bacterial inoculation and the frequency of administration), and straightforward repurposing for various types of IFN proteins.

Among the most widely prescribed protein-based drugs are IFNα and IFNβ cytokines, which are currently administered parenterally in high doses and at short intervals [66–68]. This administration regime is associated with pain and allergic reactions that reduce patient compliance and limit the use of these drugs. To overcome these drawbacks, we examined a novel delivery method for recombinant IFN_YNS_ in which the vehicle is a species of bacterium that survives the acidic conditions of the upper gastrointestinal tract to secrete IFN_YNS_ in the small intestine. To provide proof of concept, we utilized a biologically active mutant IFNα2 protein—designated IFN_YNS_—fused to a T3SS signal sequence to promote its secretion via the T3SS. We found that IFN_YNS_ was efficiently expressed and secreted, as demonstrated in Fig. 1. Furthermore, the secreted IFN_YNS_ correctly refolded and maintained its ability to activate an IFN response (Figs. 2 and 3). Moreover, the bacterially secreted IFN exhibited antiproliferative and antiviral activities similar to those of a commercially produced IFN (Figs. 4 and 5), thus suggesting that utilization of the IFN-secreting bacteria as a delivery method for oral administration of IFN is an attractive platform. Genetically modified bacteria (such as Δ*sepD*) or non-human pathogens (for example, *C. rodentium*) that secrete IFN along the GI tract offer a potentially safer alternative as it does not rely on permanent colonization. By avoiding bacterial infection while still providing the beneficial effects of IFN secretion, this approach may have significant clinical implications for treating various immune-related disorders.

Additionally, understanding the expected residence time of these bacteria within the gastrointestinal tract is essential for optimizing their efficacy and ensuring their safe use in clinical settings. Further studies are needed to evaluate the therapeutic potential of this approach entirely. Still, developing non-colonizing IFN-secreting bacteria is a promising step toward safer and more effective immunomodulation.

The proposed delivery method offers two main advantages: (i) the convenience of oral administration of IFN; and (ii) direct drug delivery to the small intestine. Nonetheless, before suggesting that oral administration can replace parenteral administration of IFN, it remains necessary to show that the drug is indeed adsorbed from the small intestine into the blood circulation. In this context, encouraging results from a recent pharmacokinetic study demonstrated that oral delivery of nanoencapsulated IFNα can produce detectable levels of IFN in the plasma [69]. Currently, IFN is administered subcutaneously, generating a high serum level of IFN, which declines rapidly with an elimination half-life of a few hours [70]. By altering the mode of administration, we envisage that the use of IFN could be broadened to treat various gastrointestinal diseases, such as enteric viral infections. This notion is based on the involvement of IFN in the antiviral defense in the gut and in the maintenance of mucosal barrier homeostasis [71, 72]. Therefore, a delivery method that generates high levels of IFN, specifically in the gut and not in the serum, might be particularly suitable for treating gastrointestinal disorders. For example, the IFN response is crucial for fighting norovirus infections, which are the leading cause of acute gastroenteritis in humans and for which there are currently no available vaccines or approved antiviral treatments [73, 74]. Indeed, the advantage of direct drug delivery to the gastrointestinal tract could be leveraged to treat various enteric diseases.

To further develop our technology, we plan to examine the oral administration of IFN in an animal model. Since the protein sequences of human and mouse IFNs and their corresponding receptors share only ~ 50% sequence identity [75], the first steps will be cloning mouse IFN and transforming the IFN-encoding plasmid into the murine-related bacterium, *C. rodentium*. This bacterium is adjusted to the mouse digestive system and contains, similarly to EPEC, a T3SS. Such a mouse model will allow us to determine IFN levels in the blood and fecal homogenates following the administration of IFN-producing bacteria and optimize the bacterial inoculum required to give the optimal plasma IFN concentration. In addition, this model system will allow us to study the immunological consequences of oral administration of IFN-producing bacteria.

## Conclusions

In the reported study, we examined, for the first time, the ability of bacteria to produce and secrete functional IFN as a potential method for oral delivery of IFN as a model protein-based drug. Our results show that this method has enormous potential for further development, particularly since it can be easily tailored to other IFN proteins.

### Supplementary Information

**Additional file 1: Figure S1.** Representative immunofluorescent images of infected and uninfected HeLa cells. HeLa cells were treated with Hoechst 33,342 dye, a DNA-specific stain of live cells, and subjected to immunofluorescent imaging to visualize virus-infected cells (these expressing GFP) among the total cell population. **Figure S2.** Representative flow cytometry plots of infected and uninfected HeLa cells. HeLa cells were stained with propidium iodide and subjected to FACS analysis to assess cell viability. The gated region corresponds to viable cells, with the percentages of viable cells provided for each condition (**A**). Histograms of GFP expression of uninfected and infected samples are presented (**B**). **Figure S3.** The bacterial supernatant of Δ*sepD* does not enhance IFNβ antiviral activity. HeLa cells were incubated with commercial IFNβ alone or IFNβ in the bacterial supernatant of Δ*sepD* EPEC for 4 h before being transduced with a GFP-expressing pseudovirus at an MOI of 1. Cells were harvested 48 h post-transduction and subjected to FACS analysis to monitor GFP expression. The results are presented as a percentage of GFP-positive cells relative to GFP-positive cells of the untreated control sample, which was not pre-incubated with bacterial supernatant. No difference between the samples was observed.

**Additional file 2: Original western-blots of experiments presented in the study**

## Data Availability

All data generated or analyzed during this study are included in this published article.

## References

[1] Platanias LC (2005). Mechanisms of type-I- and type-II-interferon-mediated signalling. Nat Rev Immunol.

[2] Kalie E, Jaitin DA, Abramovich R, Schreiber G (2007). An interferon alpha2 mutant optimized by phage display for IFNAR1 binding confers specifically enhanced antitumor activities. J Biol Chem.

[3] Levin D, Harari D, Schreiber G (2011). Stochastic receptor expression determines cell fate upon interferon treatment. Mol Cell Biol.

[4] Levin D, Schneider WM, Hoffmann HH, Yarden G, Busetto AG, Manor O (2014). Multifaceted activities of type I interferon are revealed by a receptor antagonist. Sci Signal..

[5] Filipi M, Jack S (2020). Interferons in the treatment of multiple sclerosis: a clinical efficacy, safety, and tolerability update. Int J MS Care.

[6] Ng CT, Mendoza JL, Garcia KC, Oldstone MB (2016). Alpha and beta type 1 interferon signaling: passage for diverse biologic outcomes. Cell.

[7] Runkel L, Pfeffer L, Lewerenz M, Monneron D, Yang CH, Murti A (1998). Differences in activity between alpha and beta type I interferons explored by mutational analysis. J Biol Chem.

[8] Thomas C, Moraga I, Levin D, Krutzik PO, Podoplelova Y, Trejo A (2011). Structural linkage between ligand discrimination and receptor activation by type I interferons. Cell.

[9] Tagliaferri P, Caraglia M, Budillon A, Marra M, Vitale G, Viscomi C (2005). New pharmacokinetic and pharmacodynamic tools for interferon-alpha (IFN-alpha) treatment of human cancer. Cancer Immunol Immunother.

[10] Cirelli R, Tyring SK (1995). Major therapeutic uses of interferons. Clin Immunother..

[11] Borden EC, Sen GC, Uze G, Silverman RH, Ransohoff RM, Foster GR (2007). Interferons at age 50: past, current and future impact on biomedicine. Nat Rev Drug Discov.

[12] Asmana NR (2014). Human interferon alpha-2b: a therapeutic protein for cancer treatment. Scientifica (Cairo).

[13] Sodeifian F, Nikfarjam M, Kian N, Mohamed K, Rezaei N (2022). The role of type I interferon in the treatment of COVID-19. J Med Virol.

[14] Zhou Q, Chen V, Shannon CP, Wei XS, Xiang X, Wang X (2020). Interferon-alpha2b treatment for COVID-19. Front Immunol.

[15] Sleijfer S, Bannink M, Van Gool AR, Kruit WH, Stoter G (2005). Side effects of interferon-alpha therapy. Pharm World Sci.

[16] Walther EU, Hohlfeld R (1999). Multiple sclerosis: side effects of interferon beta therapy and their management. Neurology.

[17] Deng W, Marshall NC, Rowland JL, McCoy JM, Worrall LJ, Santos AS (2017). Assembly, structure, function and regulation of type III secretion systems. Nat Rev Microbiol.

[18] Charpentier X, Oswald E (2004). Identification of the secretion and translocation domain of the enteropathogenic and enterohemorrhagic *Escherichia coli* effector Cif, using TEM-1 beta-lactamase as a new fluorescence-based reporter. J Bacteriol.

[19] Crawford JA, Kaper JB (2002). The N-terminus of enteropathogenic Escherichia coli (EPEC) Tir mediates transport across bacterial and eukaryotic cell membranes. Mol Microbiol.

[20] Munera D, Crepin VF, Marches O, Frankel G (2010). N-terminal type III secretion signal of enteropathogenic *Escherichia coli* translocator proteins. J Bacteriol.

[21] Blanco-Toribio A, Muyldermans S, Frankel G, Fernandez LA (2010). Direct injection of functional single-domain antibodies from *E*. *coli* into human cells. PLoS ONE.

[22] Guzman-Herrador DL, Fernandez-Gomez A, Llosa M (2023). Recruitment of heterologous substrates by bacterial secretion systems for transkingdom translocation. Front Cell Infect Microbiol.

[23] Azam A, Li C, Metcalf KJ, Tullman-Ercek D (2016). Type III secretion as a generalizable strategy for the production of full-length biopolymer-forming proteins. Biotechnol Bioeng.

[24] Derouazi M, Toussaint B, Quenee L, Epaulard O, Guillaume M, Marlu R (2008). High-yield production of secreted active proteins by the *Pseudomonas*
*aeruginosa* type III secretion system. Appl Environ Microbiol.

[25] Majander K, Anton L, Antikainen J, Lang H, Brummer M, Korhonen TK (2005). Extracellular secretion of polypeptides using a modified *Escherichia*
*coli* flagellar secretion apparatus. Nat Biotechnol.

[26] Reed B, Chen R (2013). Biotechnological applications of bacterial protein secretion: from therapeutics to biofuel production. Res Microbiol.

[27] Widmaier DM, Tullman-Ercek D, Mirsky EA, Hill R, Govindarajan S, Minshull J (2009). Engineering the *Salmonella* type III secretion system to export spider silk monomers. Mol Syst Biol.

[28] Deng W, Yu HB, Li Y, Finlay BB (2015). SepD/SepL-dependent secretion signals of the type III secretion system translocator proteins in enteropathogenic *Escherichia coli*. J Bacteriol.

[29] Deng W, Li Y, Hardwidge PR, Frey EA, Pfuetzner RA, Lee S (2005). Regulation of type III secretion hierarchy of translocators and effectors in attaching and effacing bacterial pathogens. Infect Immun.

[30] Portaliou AG, Tsolis KC, Loos MS, Balabanidou V, Rayo J, Tsirigotaki A (2017). Hierarchical protein targeting and secretion is controlled by an affinity switch in the type III secretion system of enteropathogenic *Escherichia*
*coli*. EMBO J.

[31] Deng W, Puente JL, Gruenheid S, Li Y, Vallance BA, Vazquez A (2004). Dissecting virulence: systematic and functional analyses of a pathogenicity island. Proc Natl Acad Sci USA.

[32] Iguchi A, Thomson NR, Ogura Y, Saunders D, Ooka T, Henderson IR (2009). Complete genome sequence and comparative genome analysis of enteropathogenic *Escherichia coli* O127:H6 strain E2348/69. J Bacteriol.

[33] Gauthier A, Puente JL, Finlay BB (2003). Secretin of the enteropathogenic *Escherichia coli* type III secretion system requires components of the type III apparatus for assembly and localization. Infect Immun.

[34] Gibson DG, Benders GA, Andrews-Pfannkoch C, Denisova EA, Baden-Tillson H, Zaveri J (2008). Complete chemical synthesis, assembly, and cloning of a *Mycoplasma*
*genitalium* genome. Science.

[35] Gibson DG, Young L, Chuang RY, Venter JC, Hutchison CA, Smith HO (2009). Enzymatic assembly of DNA molecules up to several hundred kilobases. Nat Methods.

[36] Shaulov L, Gershberg J, Deng W, Finlay BB, Sal-Man N (2017). The ruler protein EscP of the enteropathogenic *Escherichia coli* type III secretion system is involved in calcium sensing and secretion hierarchy regulation by interacting with the gatekeeper protein SepL. MBio.

[37] Tseytin I, Madar A, Mitrovic B, Deng W, Finlay BB, Sal-Man N (2018). The third transmembrane domain of EscR is critical for function of the enteropathogenic *Escherichia coli* type III secretion system. mSphere..

[38] Tseytin I, Mitrovic B, David N, Langenfeld K, Zarivach R, Diepold A (2019). The role of the small export apparatus protein, SctS, in the activity of the type III secretion system. Front Microbiol.

[39] Pfaffl MW (2001). A new mathematical model for relative quantification in real-time RT-PCR. Nucleic Acids Res.

[40] Roisman LC, Jaitin DA, Baker DP, Schreiber G (2005). Mutational analysis of the IFNAR1 binding site on IFNalpha2 reveals the architecture of a weak ligand-receptor binding-site. J Mol Biol.

[41] Kumar P, Nagarajan A, Uchil PD (2018). Analysis of cell viability by the MTT assay. Cold Spring Harb Protoc.

[42] Krasnopolsky S, Kuzmina A, Taube R (2020). Genome-wide CRISPR knockout screen identifies ZNF304 as a silencer of HIV transcription that promotes viral latency. PLoS Pathog.

[43] Baruch K, Gur-Arie L, Nadler C, Koby S, Yerushalmi G, Ben-Neriah Y (2011). Metalloprotease type III effectors that specifically cleave JNK and NF-kappaB. EMBO J.

[44] Buzzai AC, Wagner T, Audsley KM, Newnes HV, Barrett LW, Barnes S (2020). Diverse anti-tumor immune potential driven by individual IFNalpha subtypes. Front Immunol.

[45] Gessani S, Belardelli F (2021). Type I interferons as joint regulators of tumor growth and obesity. Cancers (Basel)..

[46] Gresser I, Maury C, Bandu MT, Foiret D, Trojan J, Uriel J (1984). Inhibitory effect of mouse interferon on the growth of an embryonal carcinoma in mice. J Interferon Res.

[47] Musella M, Galassi C, Manduca N, Sistigu A (2021). The Yin and Yang of type I IFNs in cancer promotion and immune activation. Biology (Basel)..

[48] Musella M, Manic G, De Maria R, Vitale I, Sistigu A (2017). Type-I-interferons in infection and cancer: unanticipated dynamics with therapeutic implications. Oncoimmunology.

[49] Sangfelt O, Erickson S, Grander D (2000). Mechanisms of interferon-induced cell cycle arrest. Front Biosci.

[50] Shi WY, Cao C, Liu L (2016). Interferon alpha induces the apoptosis of cervical cancer HeLa cells by activating both the intrinsic mitochondrial pathway and endoplasmic reticulum stress-induced pathway. Int J Mol Sci.

[51] Thyrell L, Erickson S, Zhivotovsky B, Pokrovskaja K, Sangfelt O, Castro J (2002). Mechanisms of Interferon-alpha induced apoptosis in malignant cells. Oncogene.

[52] Cordeil S, Nguyen XN, Berger G, Durand S, Ainouze M, Cimarelli A (2013). Evidence for a different susceptibility of primate lentiviruses to type I interferons. J Virol.

[53] Moraga I, Harari D, Schreiber G, Uze G, Pellegrini S (2009). Receptor density is key to the alpha2/beta interferon differential activities. Mol Cell Biol.

[54] Mundy R, MacDonald TT, Dougan G, Frankel G, Wiles S (2005). Citrobacter rodentium of mice and man. Cell Microbiol.

[55] Drucker DJ (2020). Advances in oral peptide therapeutics. Nat Rev Drug Discov.

[56] Iacob AT, Lupascu FG, Apotrosoaei M, Vasincu IM, Tauser RG, Lupascu D (2021). Recent biomedical approaches for chitosan based materials as drug delivery nanocarriers. Pharmaceutics.

[57] Yin L, Yuvienco C, Montclare JK (2017). Protein based therapeutic delivery agents: contemporary developments and challenges. Biomaterials.

[58] Lynch JP, Gonzalez-Prieto C, Reeves AZ, Bae S, Powale U, Godbole NP (2023). Engineered *Escherichia*
*coli* for the in situ secretion of therapeutic nanobodies in the gut. Cell Host Microbe.

[59] Pendergrass HA, May AE (2020). Delivery of heterologous proteins, enzymes, and antigens via the bacterial type III secretion system. Microorganisms..

[60] Russmann H, Shams H, Poblete F, Fu Y, Galan JE, Donis RO (1998). Delivery of epitopes by the *Salmonella* type III secretion system for vaccine development. Science.

[61] Verma NK, Ziegler HK, Wilson M, Khan M, Safley S, Stocker BA (1995). Delivery of class I and class II MHC-restricted T-cell epitopes of listeriolysin of *Listeria monocytogenes* by attenuated *Salmonella*. Vaccine.

[62] Wieser A, Magistro G, Norenberg D, Hoffmann C, Schubert S (2012). First multi-epitope subunit vaccine against extraintestinal pathogenic *Escherichia coli* delivered by a bacterial type-3 secretion system (T3SS). Int J Med Microbiol.

[63] Metcalf KJ, Bevington JL, Rosales SL, Burdette LA, Valdivia E, Tullman-Ercek D (2016). Proteins adopt functionally active conformations after type III secretion. Microb Cell Fact.

[64] Lim D, Jung WC, Jeong JH, Song M (2020). Targeted delivery of the mitochondrial target domain of noxa to tumor tissue via synthetic secretion system in *E*. *coli*. Front Bioeng Biotechnol..

[65] Tyurin AA, Kabardaeva KV, Mustafaev ON, Pavlenko OS, Sadovskaya NS, Fadeev VS (2018). Expression of soluble active interferon alphaA in *Escherichia coli* periplasm by fusion with thermostable lichenase using the domain insertion approach. Biochemistry (Mosc).

[66] Hauser SL, Bar-Or A, Comi G, Giovannoni G, Hartung HP, Hemmer B (2017). Ocrelizumab versus interferon beta-1a in relapsing multiple sclerosis. N Engl J Med.

[67] Lazear HM, Schoggins JW, Diamond MS (2019). Shared and distinct functions of type I and type III interferons. Immunity.

[68] Phillips S, Mistry S, Riva A, Cooksley H, Hadzhiolova-Lebeau T, Plavova S (2017). Peg-interferon lambda treatment induces robust innate and adaptive immunity in chronic Hepatitis B patients. Front Immunol.

[69] Canepa C, Imperiale JC, Berini CA, Lewicki M, Sosnik A, Biglione MM (2017). Development of a drug delivery system based on chitosan nanoparticles for oral administration of interferon-alpha. Biomacromol.

[70] Wills RJ (1990). Clinical pharmacokinetics of interferons. Clin Pharmacokinet.

[71] Mahlakoiv T, Hernandez P, Gronke K, Diefenbach A, Staeheli P (2015). Leukocyte-derived IFN-alpha/beta and epithelial IFN-lambda constitute a compartmentalized mucosal defense system that restricts enteric virus infections. PLoS Pathog.

[72] McElrath C, Espinosa V, Lin JD, Peng J, Sridhar R, Dutta O (2021). Critical role of interferons in gastrointestinal injury repair. Nat Commun.

[73] Roth AN, Karst SM (2016). Norovirus mechanisms of immune antagonism. Curr Opin Virol.

[74] Tan M (2021). Norovirus vaccines: current clinical development and challenges. Pathogens.

[75] Harari D, Abramovich R, Zozulya A, Smith P, Pouly S, Koster M (2014). Bridging the species divide: transgenic mice humanized for type-I interferon response. PLoS ONE.

[76] Schauer DB, Falkow S (1993). The eae gene of Citrobacter freundii biotype 4280 is necessary for colonization in transmissible murine colonic hyperplasia. Infect Immun.

