# Supplementary material for: Secretion of functional interferon by the type 3 secretion system of enteropathogenic Escherichia coli
Source: Microb Cell Fact. 2024 Jun 1;23:163. doi: 10.1186/s12934-024-02397-y (PMC11144349; doi:10.1186/s12934-024-02397-y)
Supplement: Supplementary file 1 — Additional file 1: Figure S1. Representative immunofluorescent images of infected and uninfected HeLa cells. HeLa cells were treated with Hoechst 33,342 dye, a DNA-specific stain of live cells, and subjected to immunofluorescent imaging to visualize virus-infected cells (these expressing GFP) among the total cell population. Figure S2. Representative flow cytometry plots of infected and uninfected HeLa cells. HeLa cells were stained with propidium iodide and subjected to FACS analysis to assess cell viability. The gated region corresponds to viable cells, with the percentages of viable cells provided for each condition (A). Histograms of GFP expression of uninfected and infected samples are presented (B). Figure S3. The bacterial supernatant of ΔsepD does not enhance IFNβ antiviral activity. HeLa cells were incubated with commercial IFNβ alone or IFNβ in the bacterial supernatant of ΔsepD EPEC for 4 h before being transduced with a GFP-expressing pseudovirus at an MOI of 1. Cells were harvested 48 h post-transduction and subjected to FACS analysis to monitor GFP expression. The results are presented as a percentage of GFP-positive cells relative to GFP-positive cells of the untreated control sample, which was not pre-incubated with bacterial supernatant. No difference between the samples was observed. [file 12934_2024_2397_MOESM1_ESM.docx]

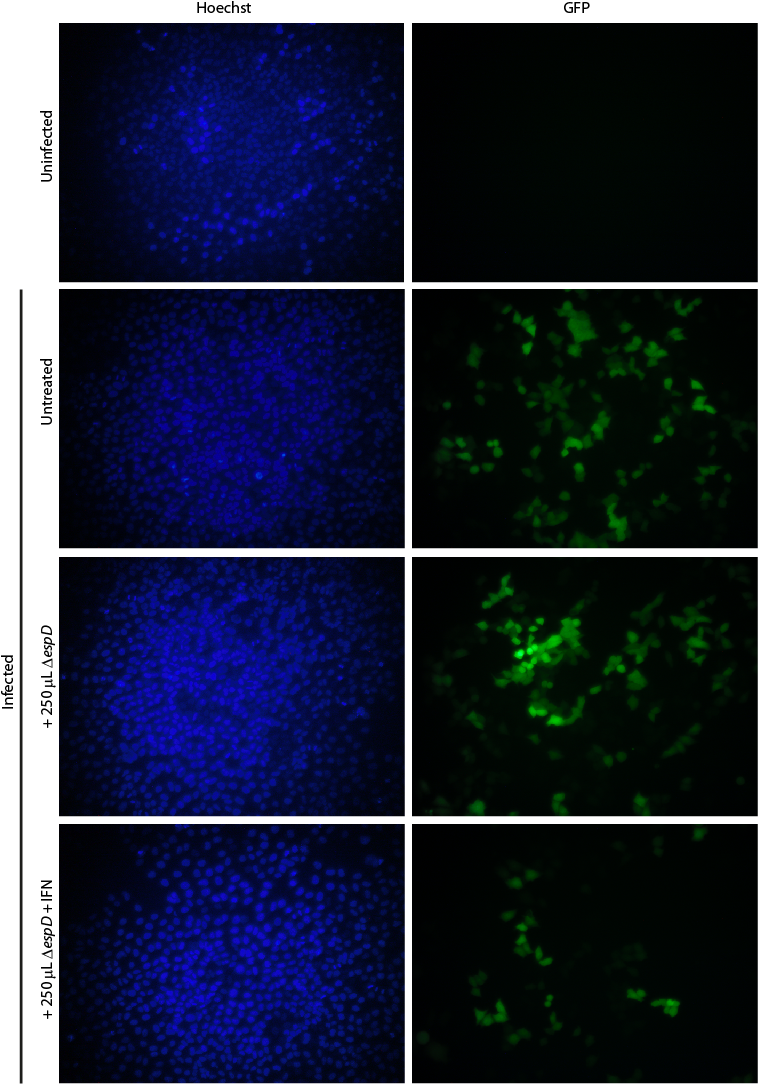


**Figure S1:** **Representative immunofluorescent images of infected and uninfected HeLa cells.** HeLa cells were treated with Hoechst 33342 dye, a DNA-specific stain of live cells, and subjected to immunofluorescent imaging to visualize virus-infected cells (these expressing GFP) among the total cell population.


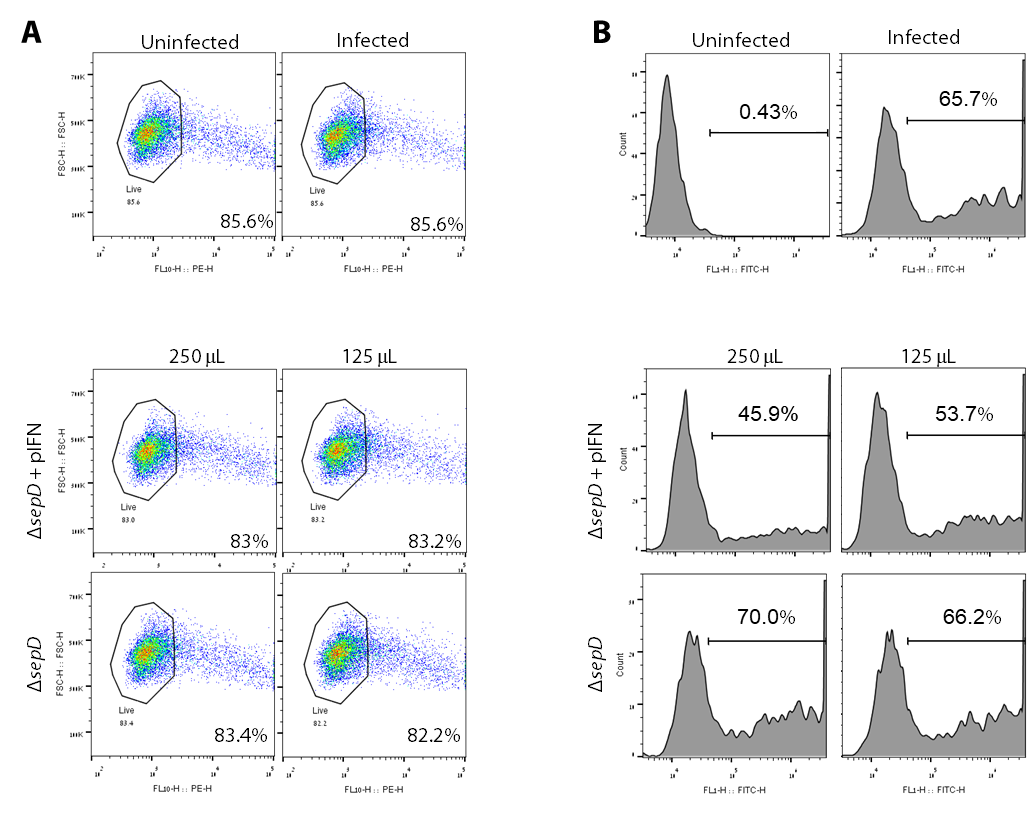


**Figure S2: Representative flow cytometry plots of infected and uninfected HeLa cells.** HeLa cells were stained with propidium iodide and subjected to FACS analysis to assess cell viability. The gated region corresponds to viable cells, with the percentages of viable cells provided for each condition (A). Histograms of GFP expression of uninfected and infected samples are presented (B).


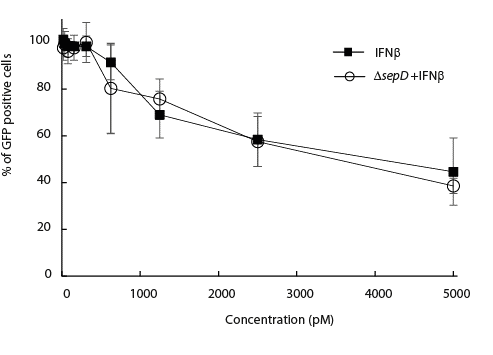


**Figure S3:** **The bacterial supernatant of Δ*sepD* does not enhance IFNβ antiviral activity**. HeLa cells were incubated with commercial IFNβ alone or IFNβ in the bacterial supernatant of Δ*sepD* EPEC for 4 hours before being transduced with a GFP-expressing pseudovirus at an MOI of 1. Cells were harvested 48 hours post-transduction and subjected to FACS analysis to monitor GFP expression. The results are presented as a percentage of GFP-positive cells relative to GFP-positive cells of the untreated control sample, which was not pre-incubated with bacterial supernatant. No difference between the samples was observed.
