# Supplementary material for: Secretion of functional interferon by the type 3 secretion system of enteropathogenic Escherichia coli
Source: Microb Cell Fact. 2024 Jun 1;23:163. doi: 10.1186/s12934-024-02397-y (PMC11144349; doi:10.1186/s12934-024-02397-y)

Original WB gels:

**Figure 1:**

Supernatants

Coomassie:


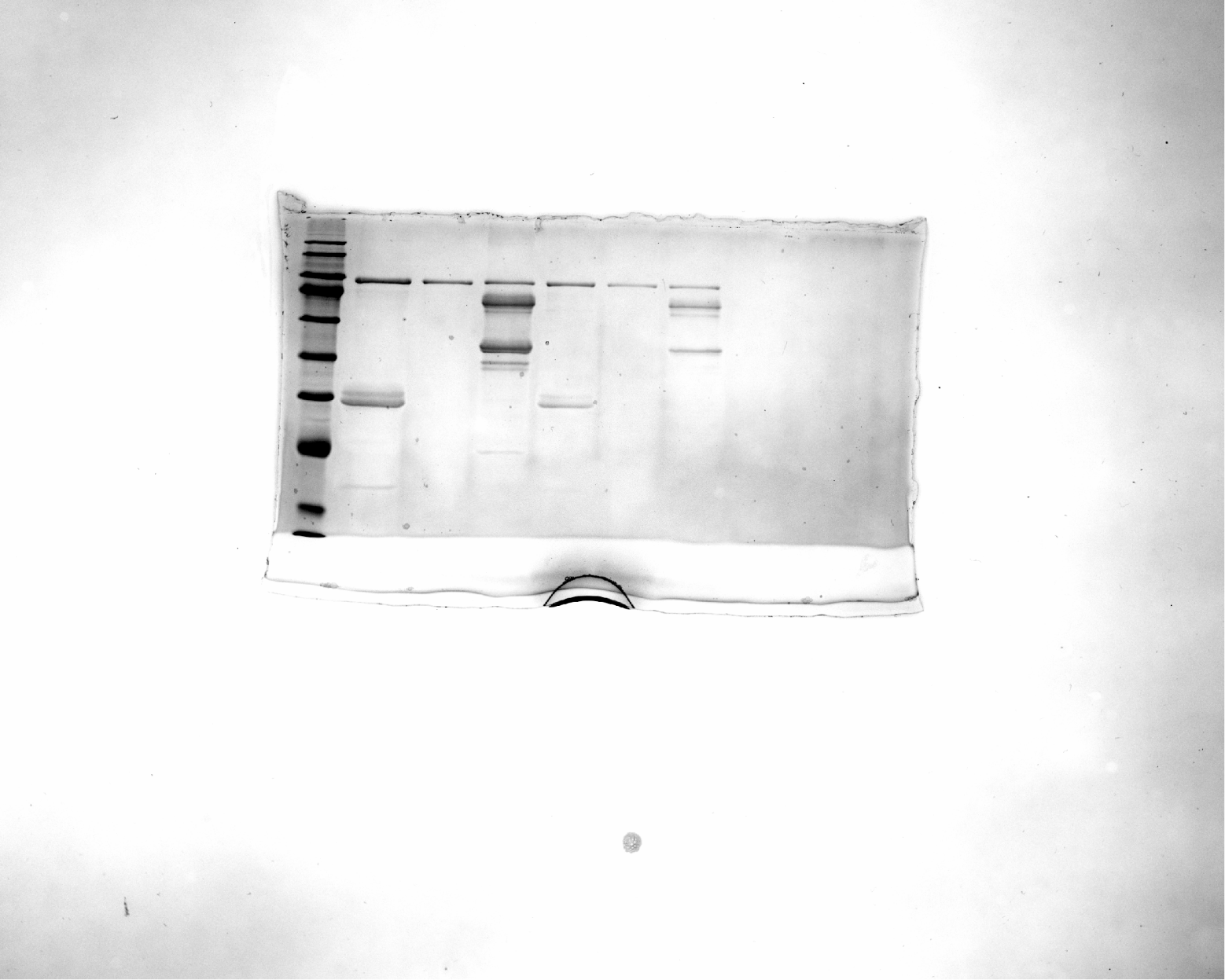


Anti Tir:


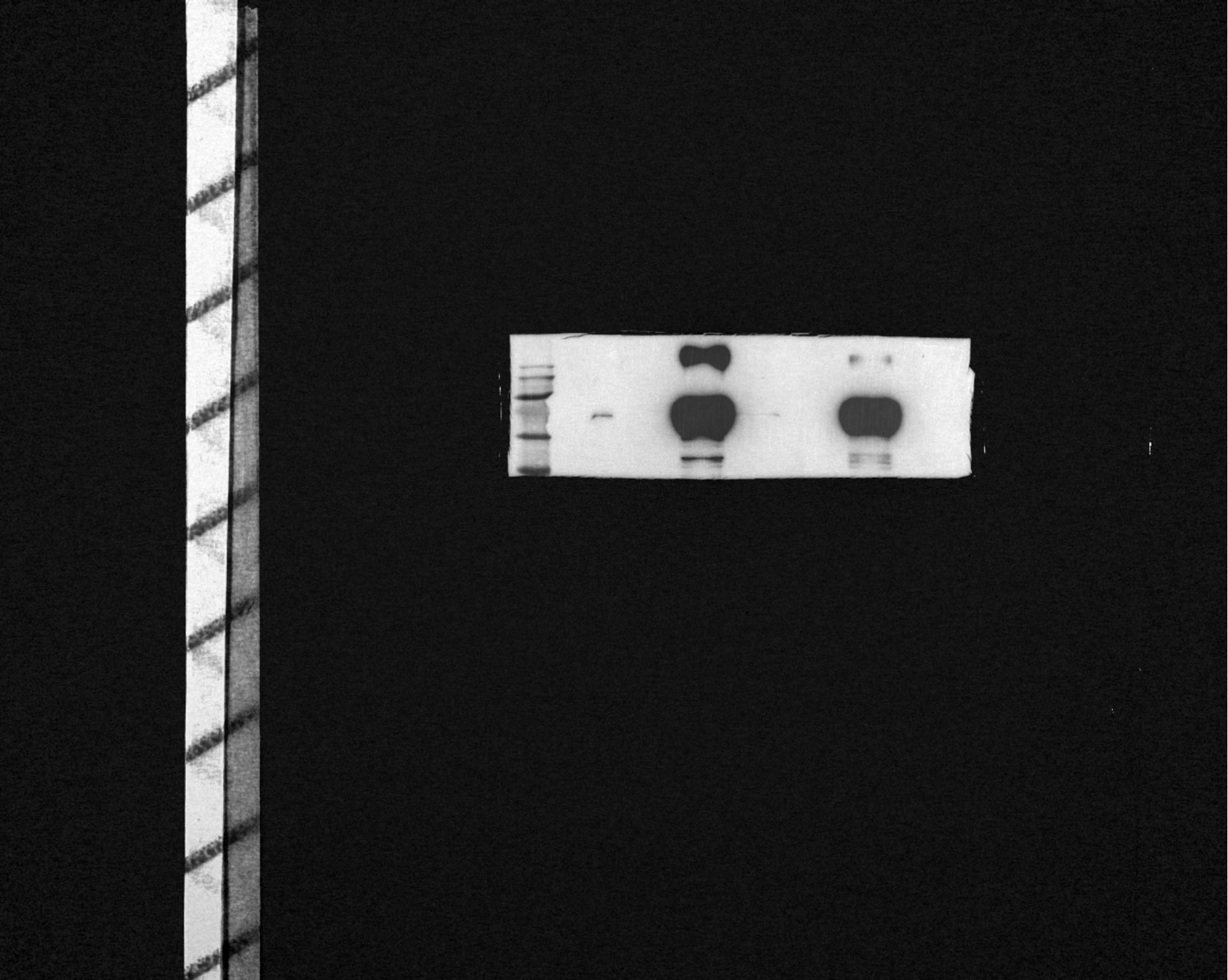


anti-IFN:


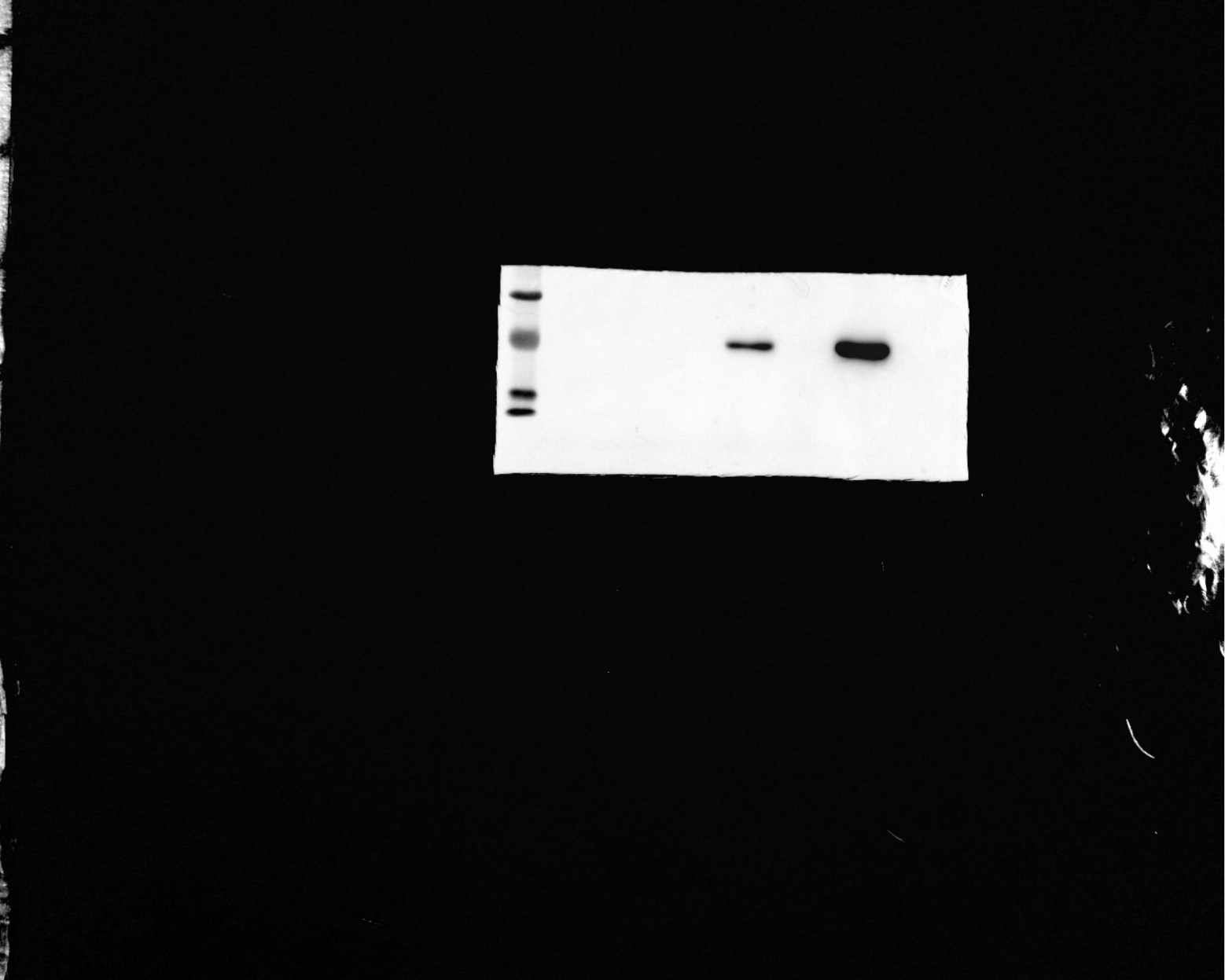


Pellets:

anti-IFN:


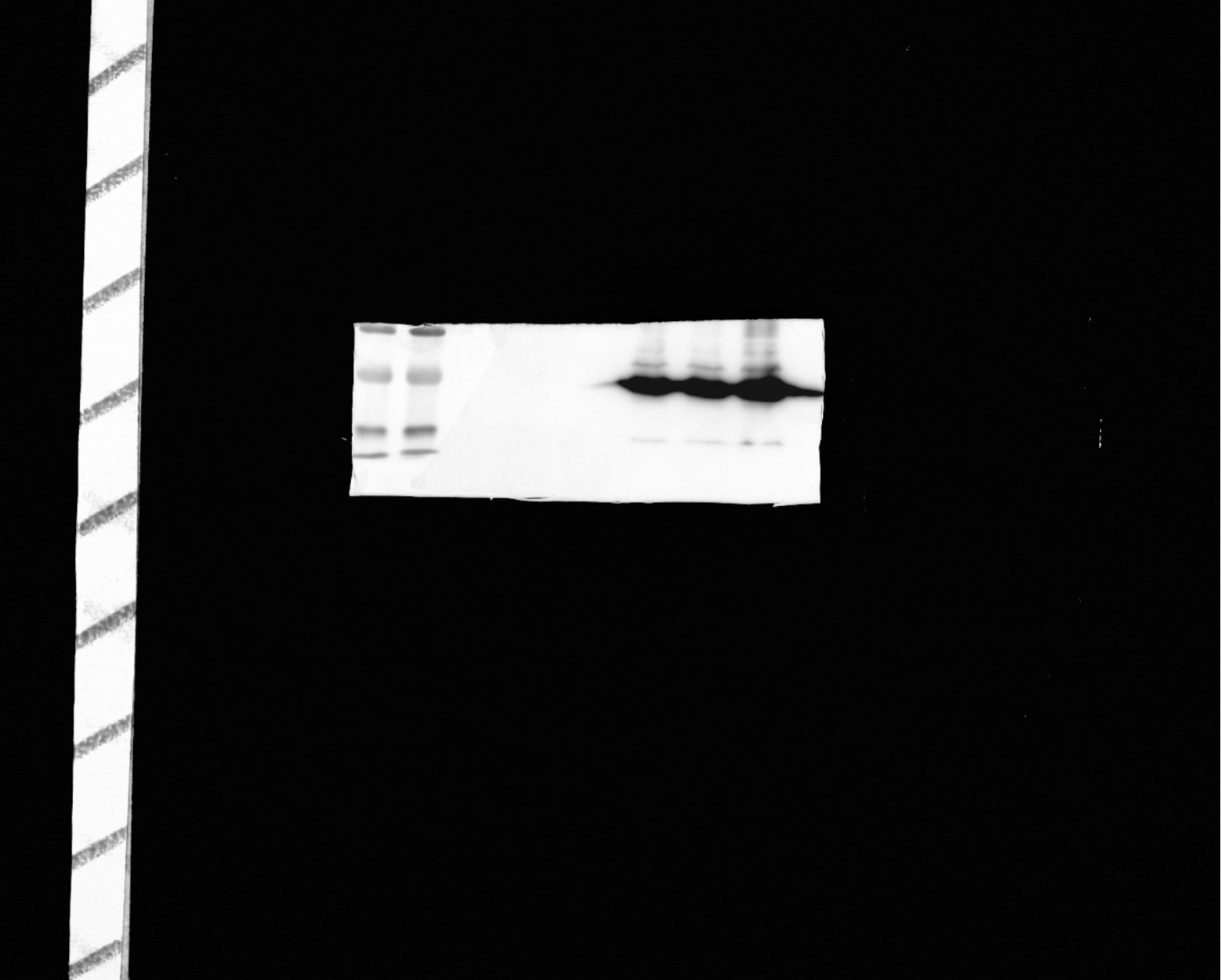


Anti-DnaK:


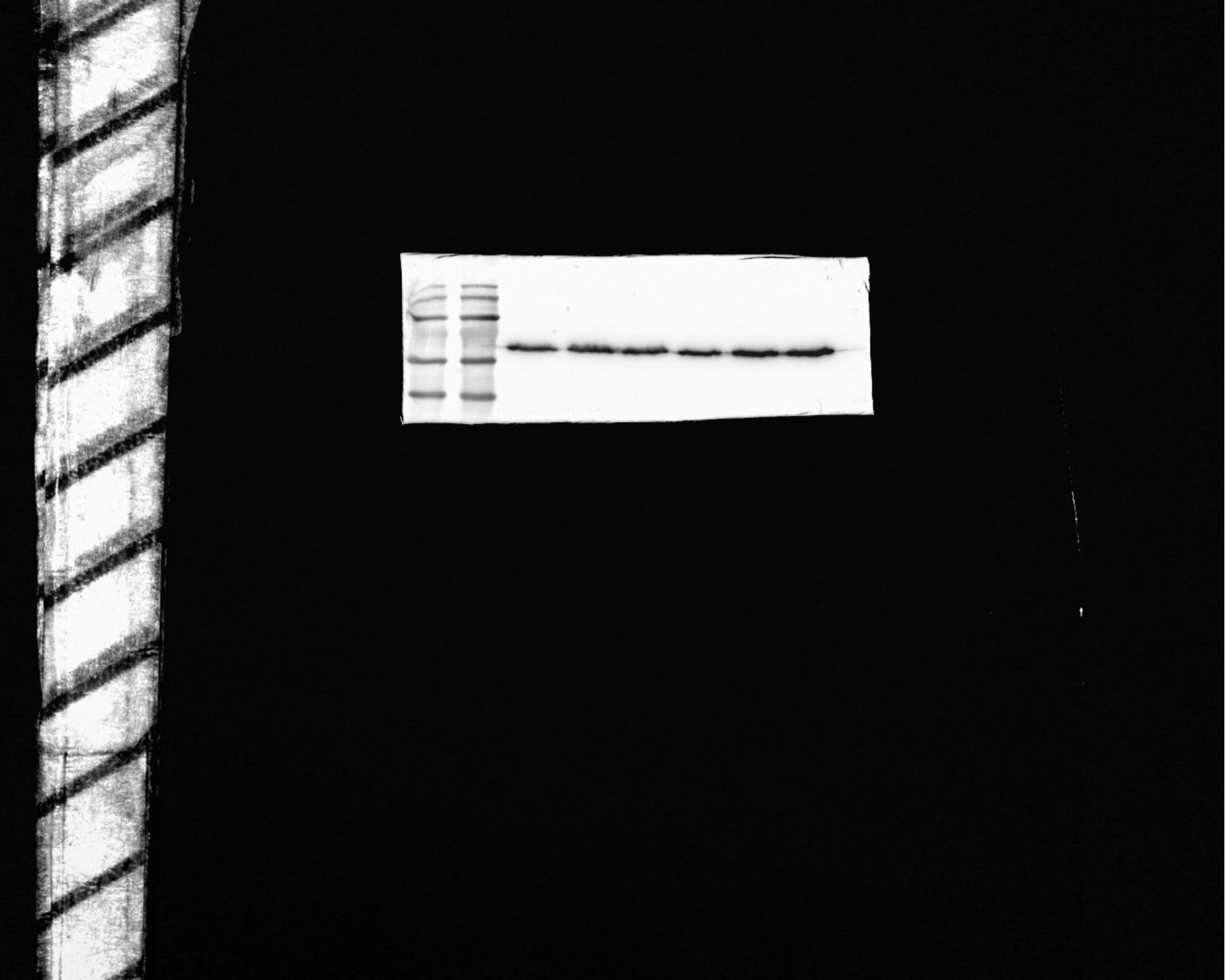


**Figure 2:**

**A**: Aerobic conditions: The membrane was cut on the 60 kDa marker. The upper part was incubated with an anti-phospho-STAT antibody, and the lower with an anti-actin antibody.


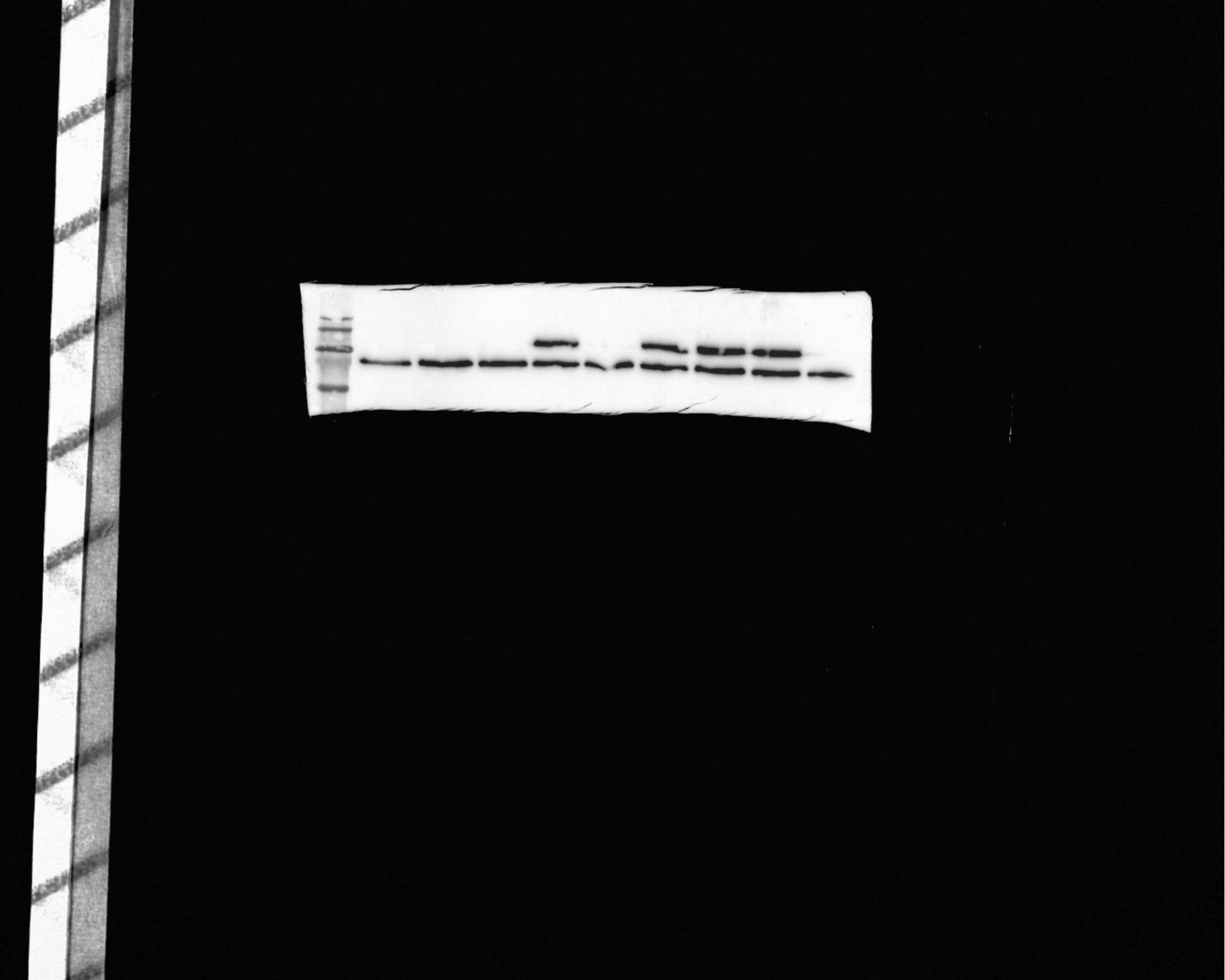


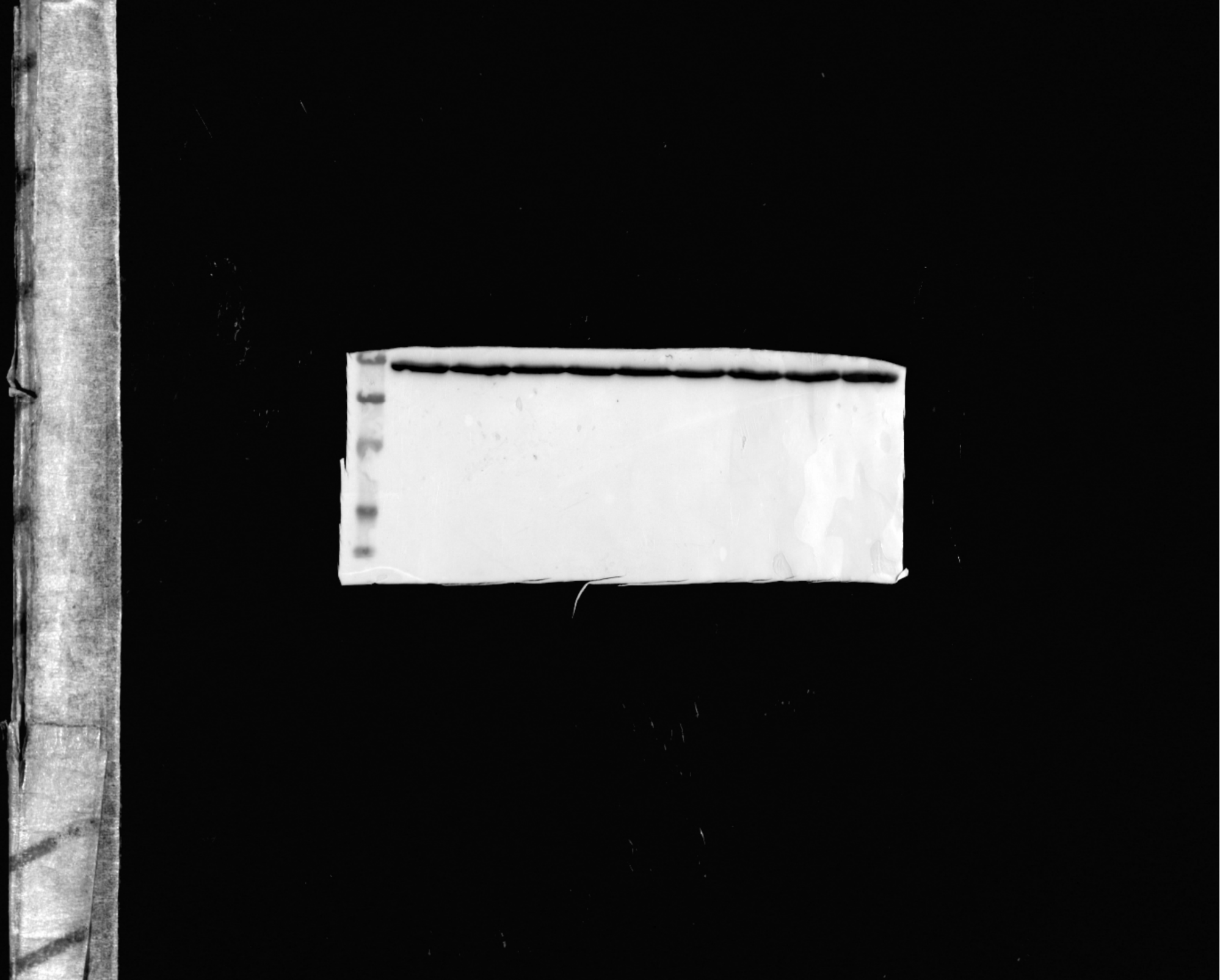


**B**: Anaerobic conditions: Upper blot - anti-phospho-STAT antibody, lower blot - anti-actin antibody.


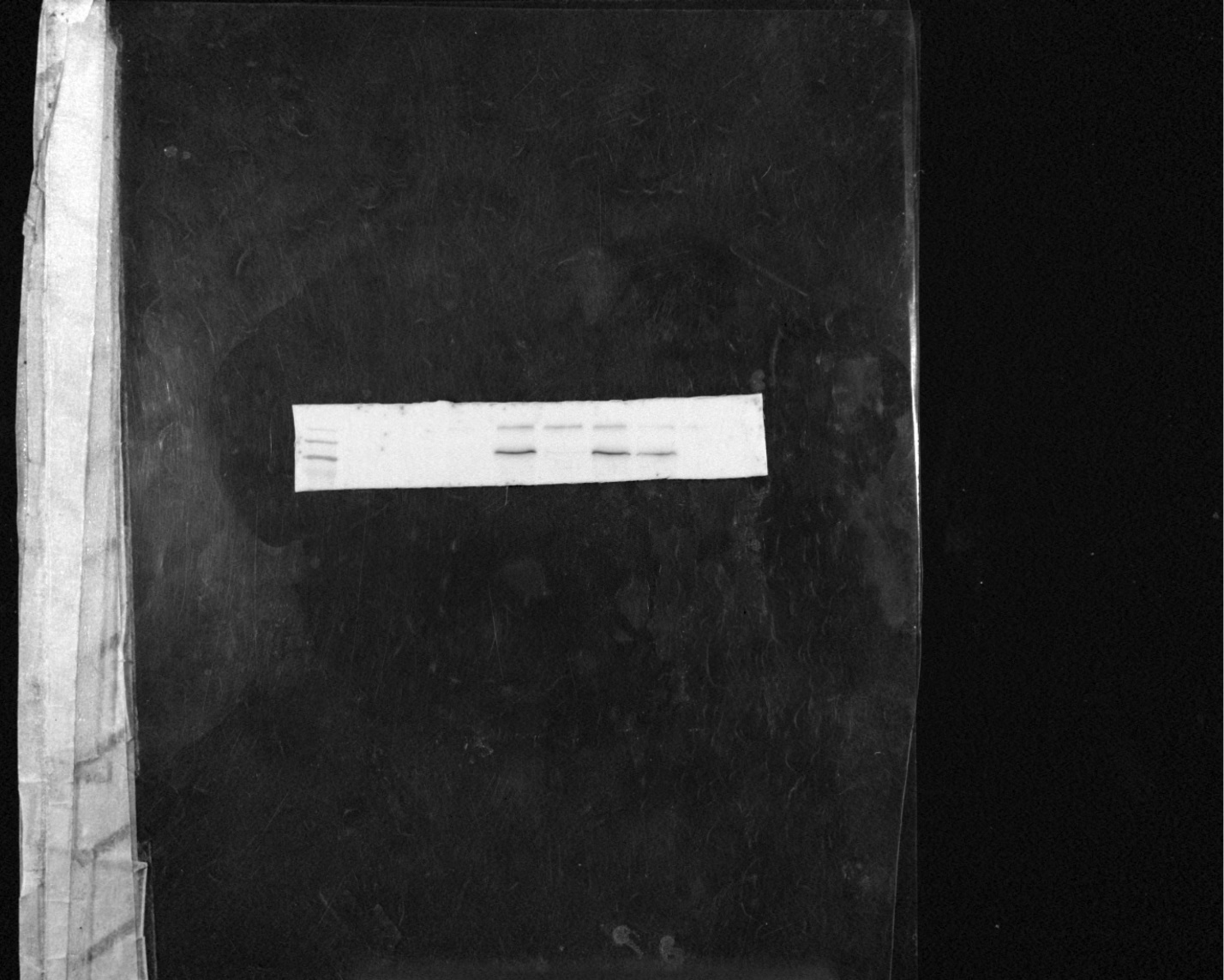


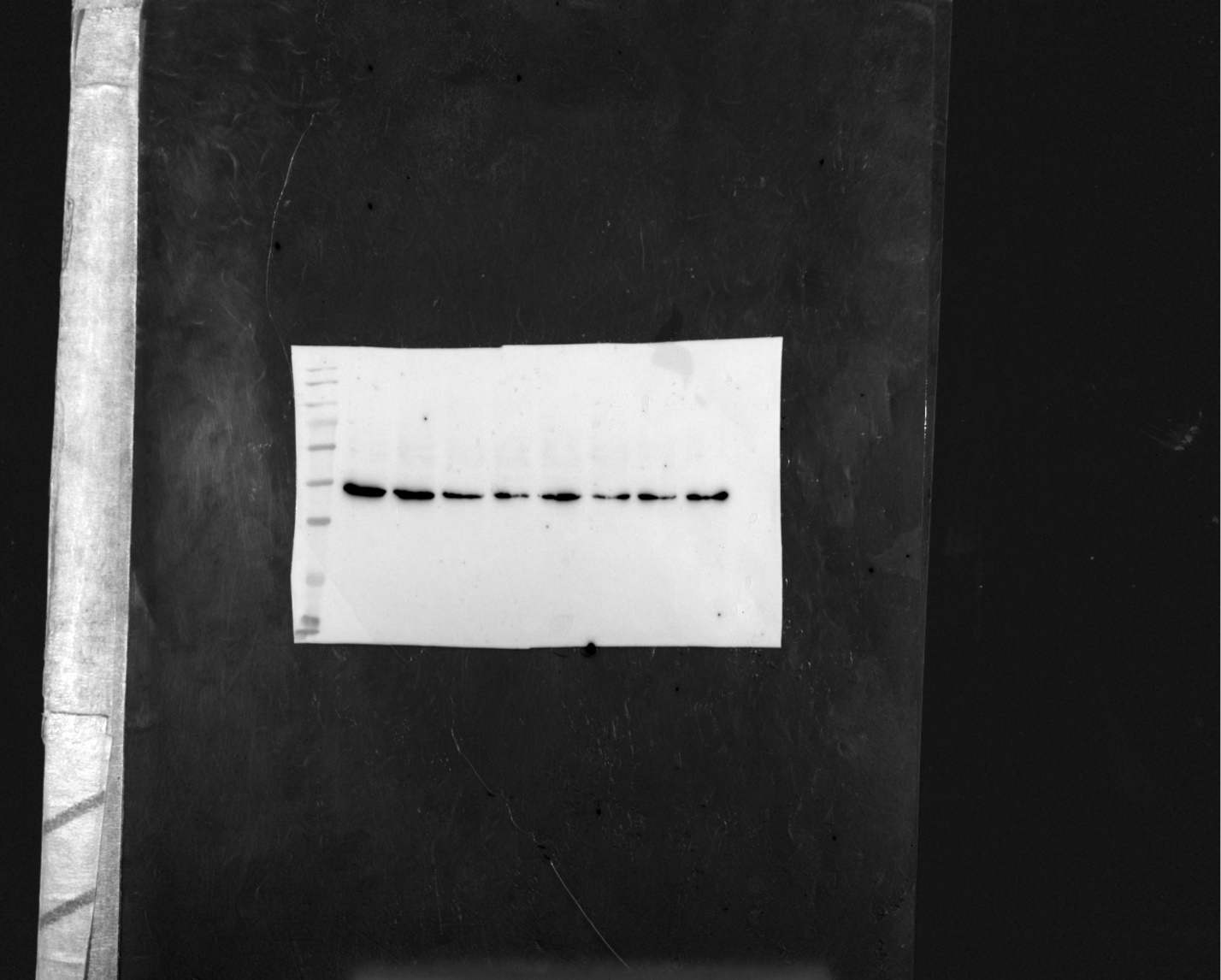


**C**: The Blot was cut on the 60 kDa marker. The upper part was incubated with anti-phospho-STAT, and the lower part with anti-actin antibody.


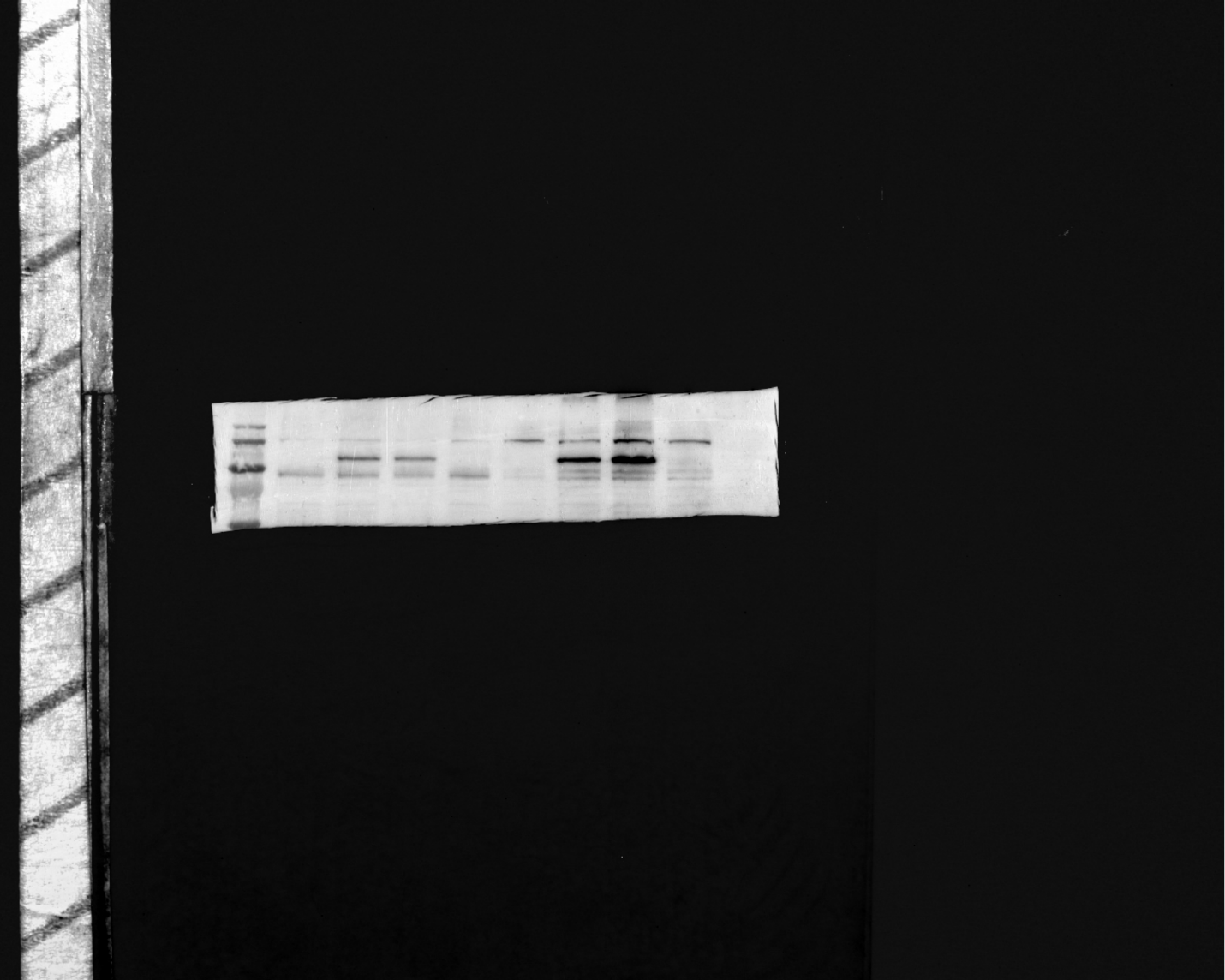


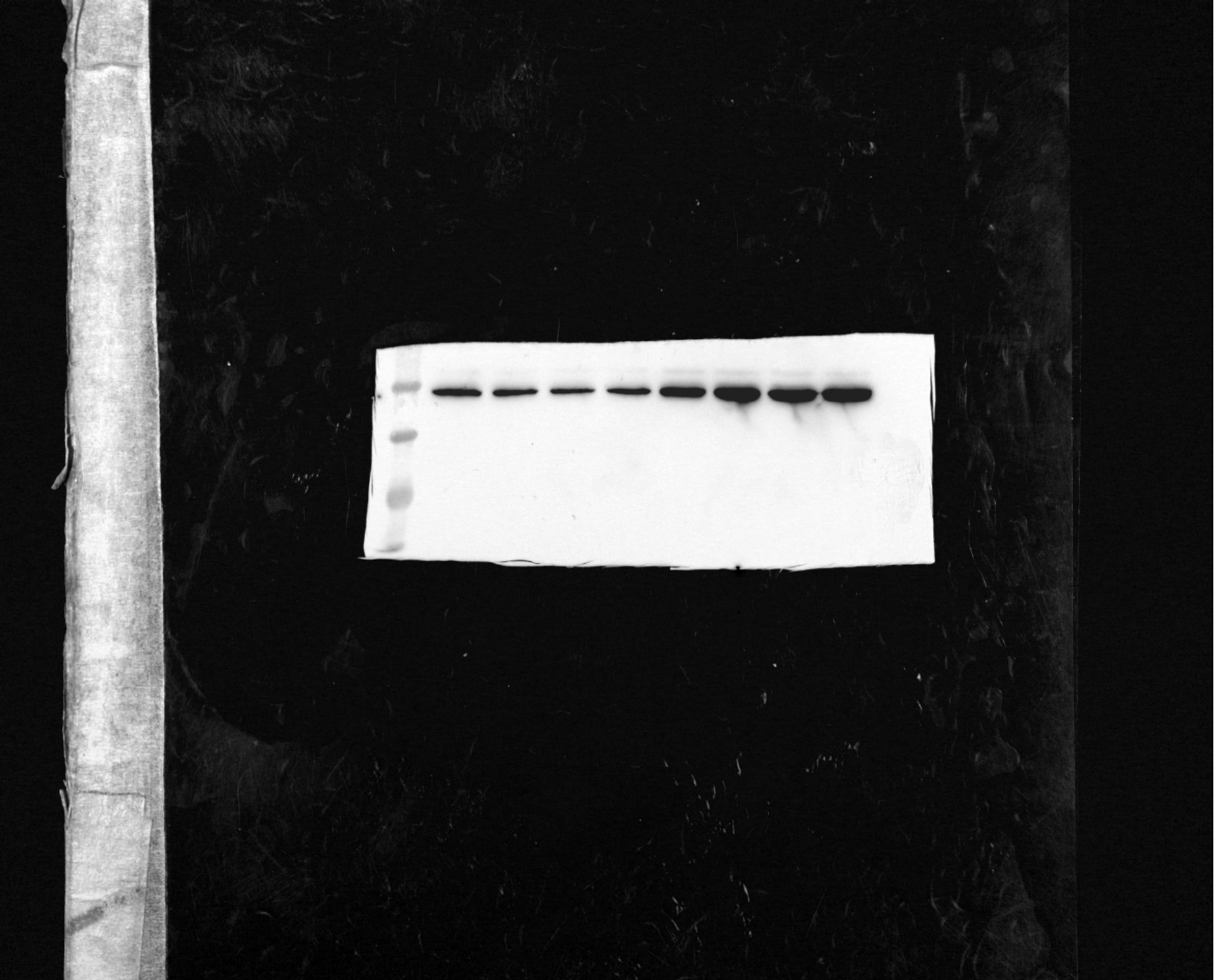


**
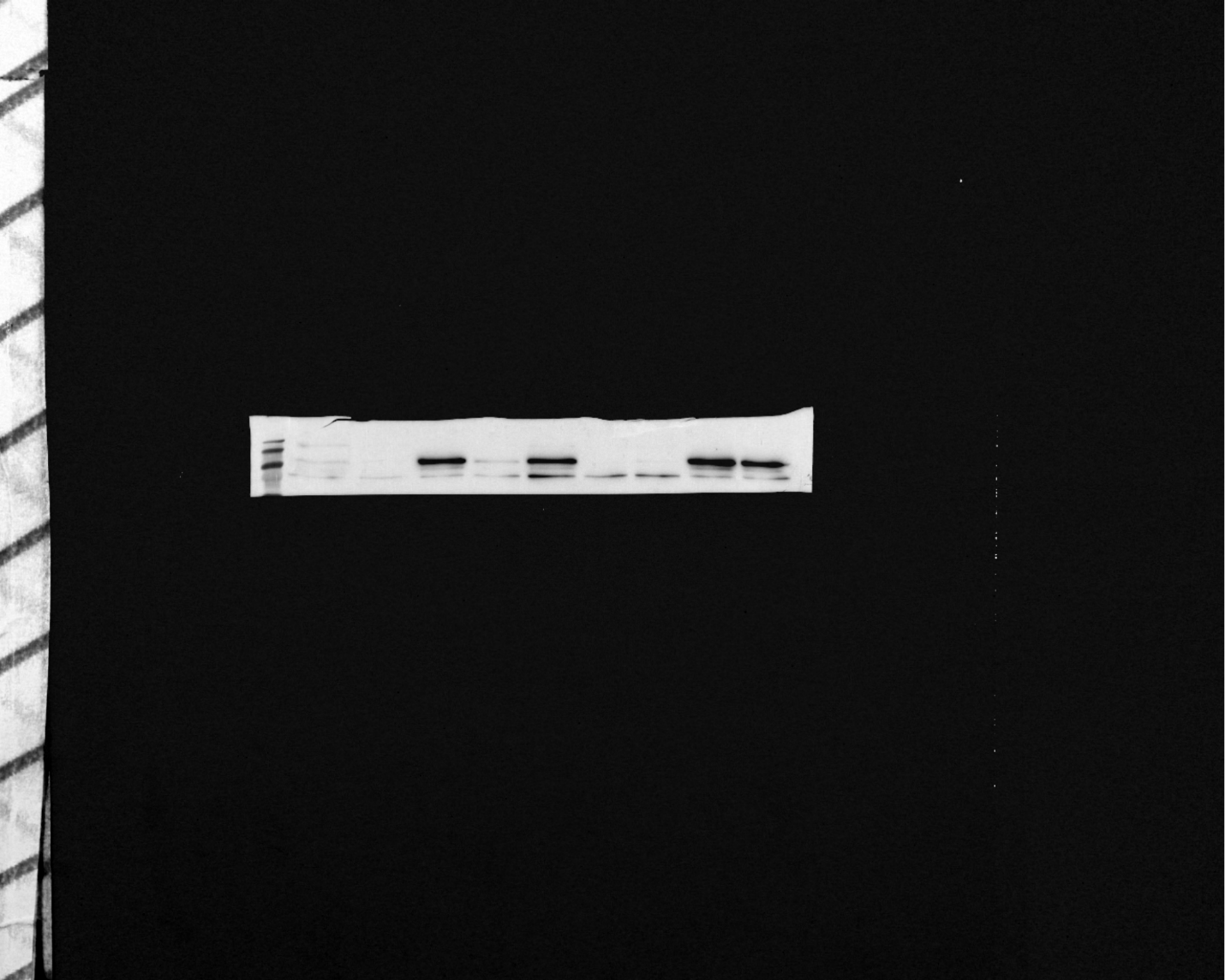
D**: Neutralization assay. The blot was cut on the 60 kDa marker. The upper part was incubated with anti-phospho-STAT, and the lower part with anti-actin antibody.


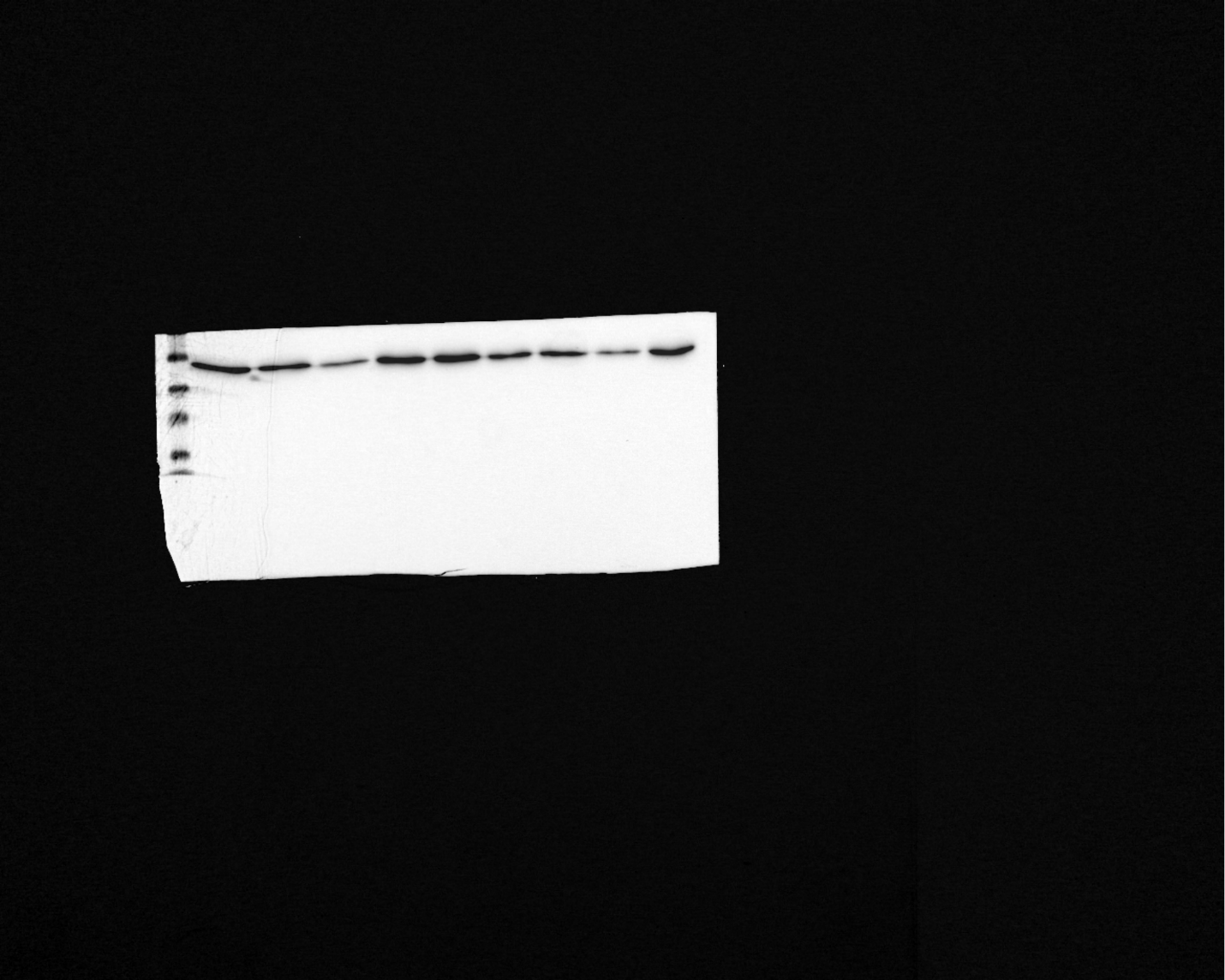


**Figure 6:**

**A**: Direct infection. The blot was cut on the 60 kDa marker. The upper part was incubated with anti-phospho-STAT, and the lower part with anti-actin antibody.


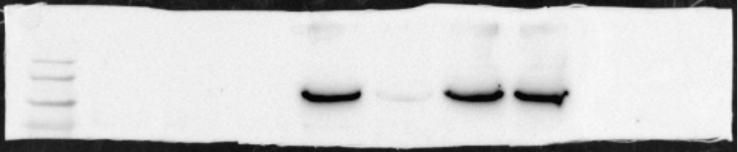


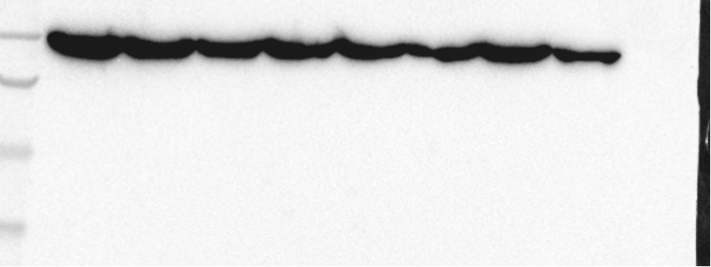


**B**: The upper gel was incubated with an anti-JNK antibody, and the lower gel was incubated with an anti-actin antibody.


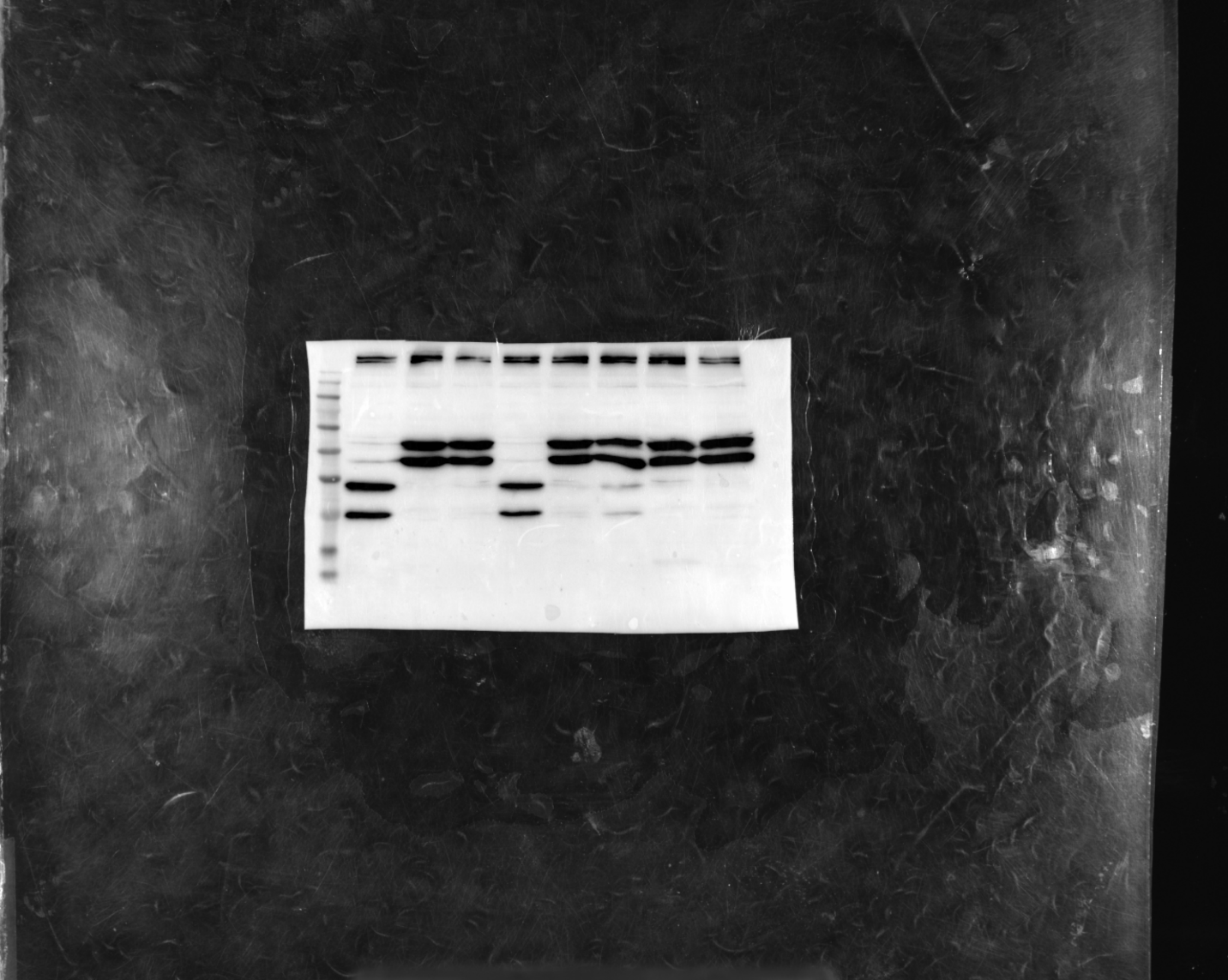


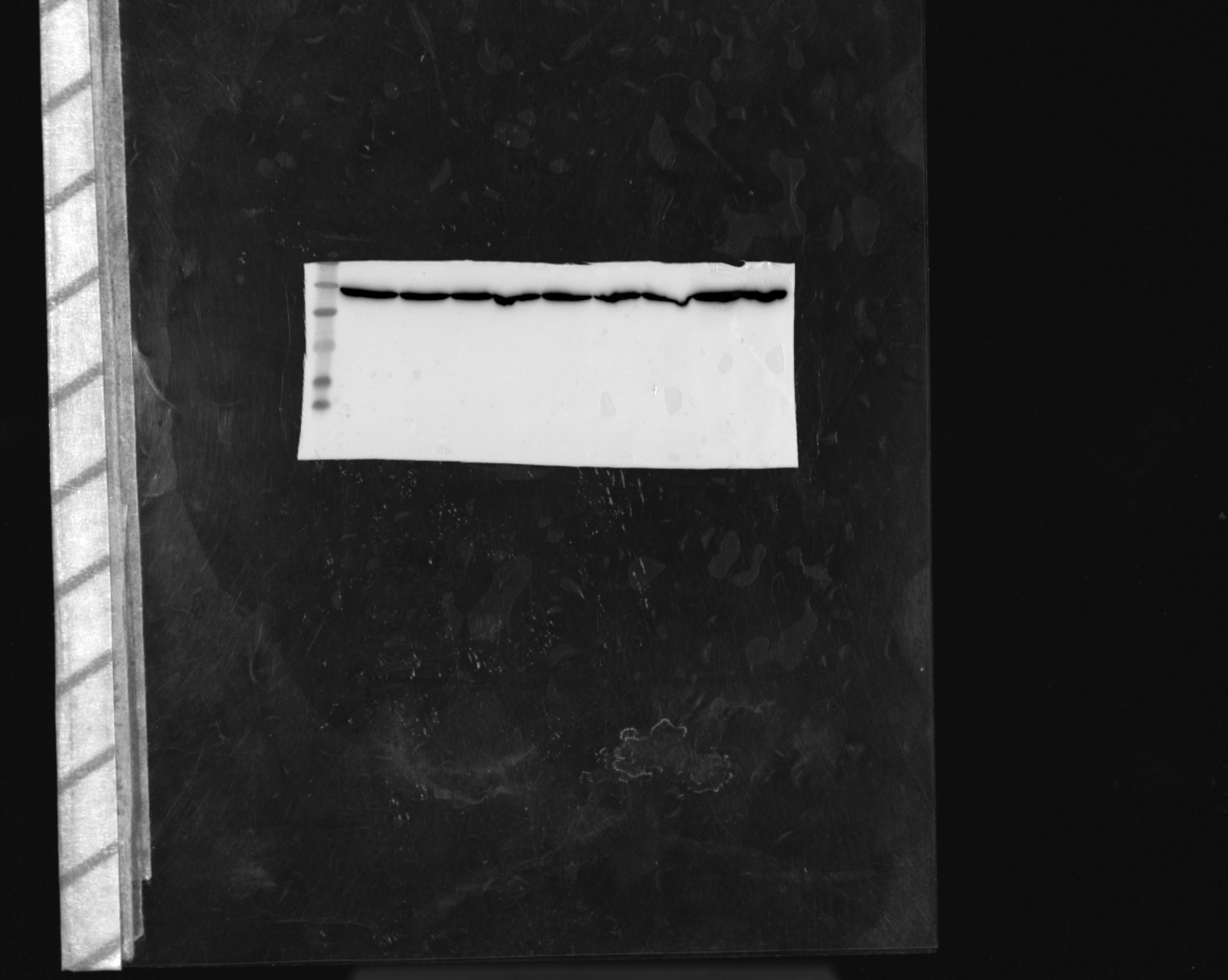


**Figure 7**:

**A**: Secretion of IFN (anti-IFN antibody) when expressed in *Citrobacter rodentium*. The gel shows two repeats (the left section is presented in the manuscript).


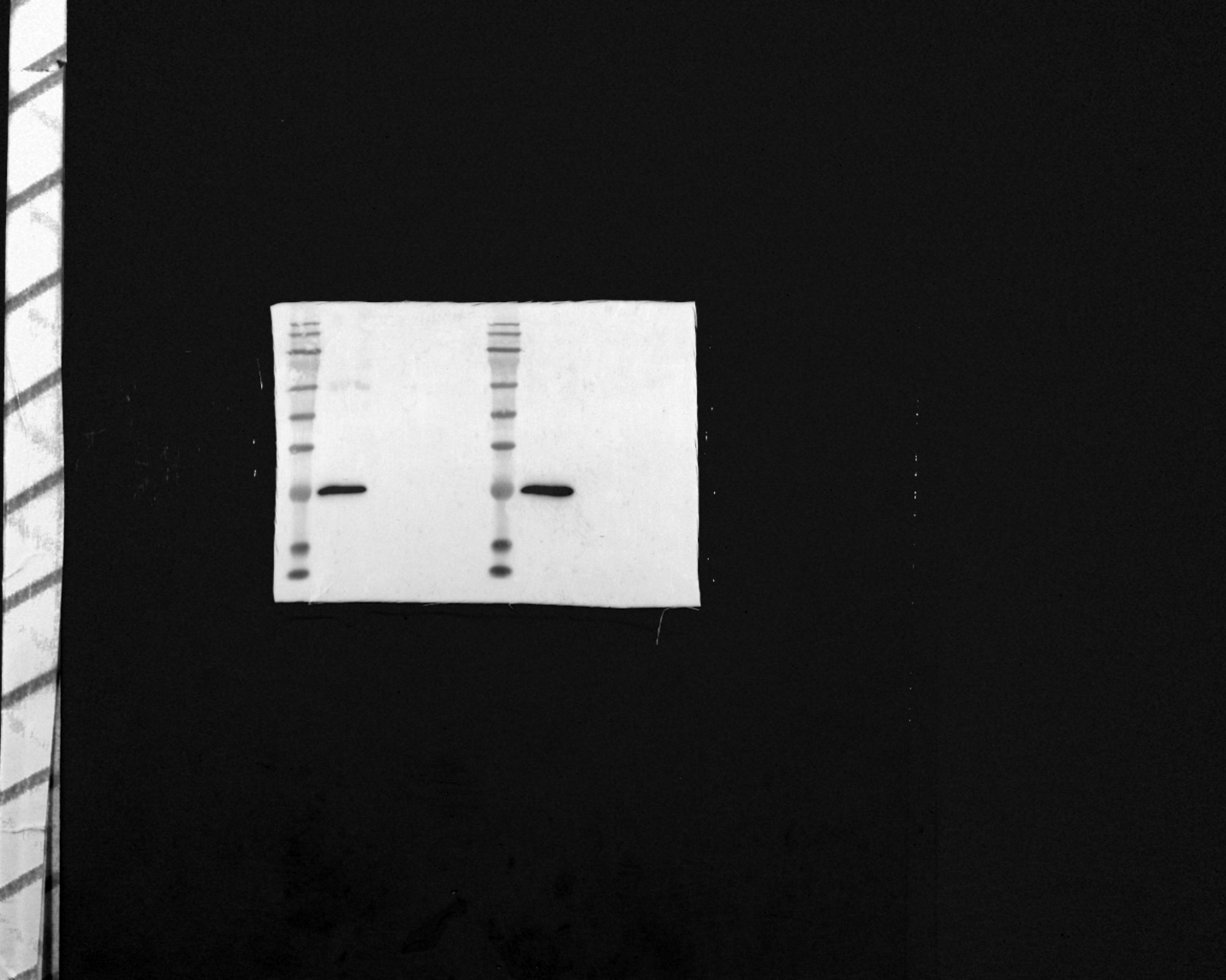


**B**: pSTAT activation with supernatants of *C. rodentium*. The blot was cut on the 60 kDa marker. The upper part was incubated with anti-phospho-STAT, and the lower part with anti-actin antibody.


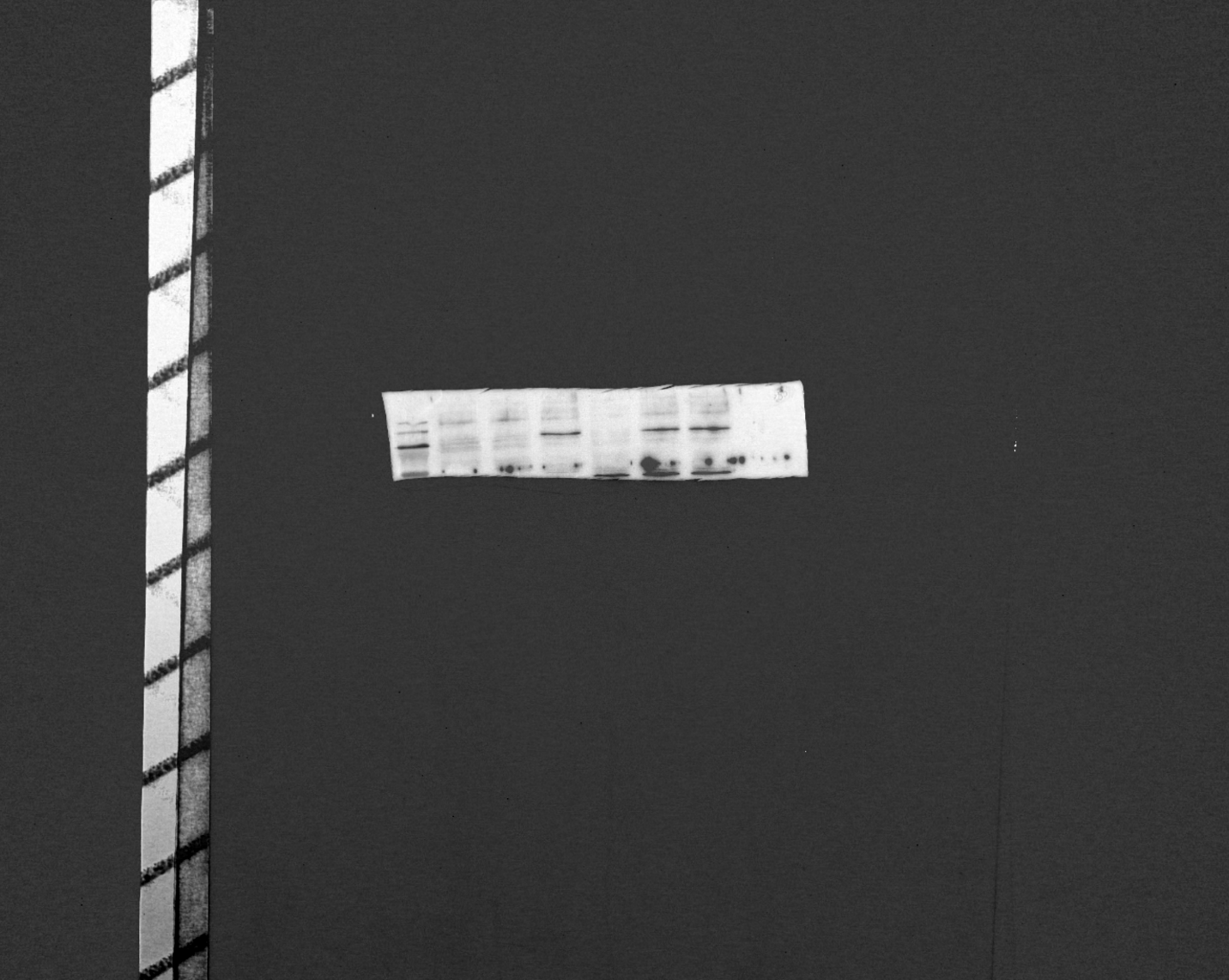


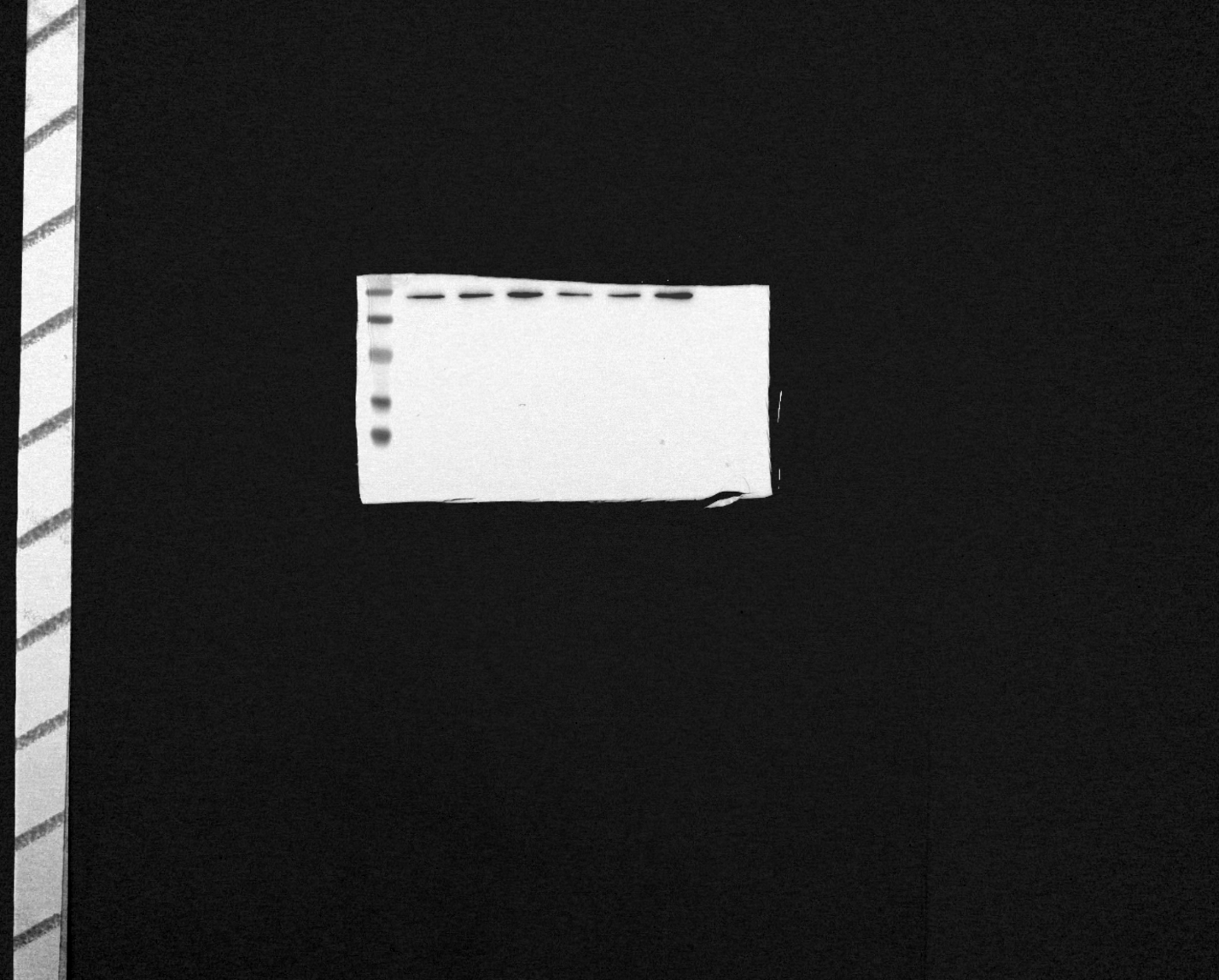

Supplement: Supplementary file 2 — Additional file 2: Original western-blots of experiments presented in the study [file 12934_2024_2397_MOESM2_ESM.docx]
